# Epigenetic Alterations in Age-Related Macular Degeneration: Mechanisms and Implications

**DOI:** 10.3390/ijms26157601

**Published:** 2025-08-06

**Authors:** Dana Kisswani, Christina Carroll, Fatima Valdes-Mora, Matt Rutar

**Affiliations:** 1 Faculty of Science and Technology, University of Canberra, Canberra, ACT 2617, Australia; 2 The John Curtin School of Medical Research, The Australian National University, Canberra, ACT 2601, Australia; 3 Cancer Epigenetic Biology and Therapeutics, Therapeutic Discovery Theme, Children’s Cancer Institute, Sydney, NSW 2033, Australia; 4 School of Clinical Medicine, Faculty of Medicine & Health, UNSW Sydney, Sydney, NSW 2033, Australia; 5 School of Biomedical Engineering, Faculty of Engineering and IT, University of Technology Sydney, Ultimo, NSW 2007, Australia; 6 Department of Anatomy and Physiology, University of Melbourne, Melbourne, VIC 3052, Australia

**Keywords:** age-related macular degeneration, chromatin accessibility, DNA methylation, epigenetics, histone acetylation, histone methylation, histone modifications, histone variants, long noncoding RNA

## Abstract

Age-related macular degeneration (*AMD*) is one of the leading causes of irreversible vision loss among the elderly, and is influenced by a combination of genetic and environmental risk factors. While genetic associations in AMD are well-established, the molecular mechanisms underlying disease onset and progression remain poorly understood. A growing body of evidence suggests that epigenetic modifications may serve as a potential missing link regulating gene–environment interactions. This review incorporates recent findings on DNA methylation, including both hypermethylation and hypomethylation patterns affecting genes such as silent mating type information regulation 2 homolog 1 (*SIRT1*), glutathione S-transferase isoform (*GSTM*), and SKI proto-oncogene (*SKI*), which may influence key pathophysiological drivers of AMD. We also examine histone modification patterns, chromatin accessibility, the status of long non-coding RNAs (*lncRNAs*) in AMD pathogenesis and in regulating pathways pertinent to the pathophysiology of the disease. While the field of ocular epigenetics remains in its infancy, accumulating evidence to date points to a burgeoning role for epigenetic regulation in AMD, pre-clinical studies have yielded promising findings for the prospect of epigenetics as a future therapeutic avenue.

## 1. Introduction

Age-related macular degeneration (AMD) is the leading cause of vision loss in developed countries, affecting approximately 10% of individuals over the age of 65 and 25% of those over 75 [1,2]. The global prevalence of AMD has been estimated to reach 288 million by 2040, from a reported 196 million in 2020, underscoring the current unmet need for improved therapeutic targets and strategies [3]. AMD affects key structures in the eye, including photoreceptors, the retinal pigment epithelium (RPE), Bruch’s membrane (BrM), and the choroid [4]. Damage is most pronounced in the macula, the region responsible for central vision, high visual acuity, and color perception [4,5]. Consequently, people living with AMD struggle with everyday tasks such as reading, facial recognition, and driving [5].

AMD is a multifactorial disease, shaped by both genetic and lifestyle factors [4]. A clear role of genetics was crystallized in the early 2000s, when variants in the complement factor H (*CFH*) gene were identified as significant risk alleles via genome-wide association studies (GWAS) [6,7,8]. *CFH* encodes a regulatory protein of the complement cascade, a crucial innate immune pathway, and its dysregulation has been suggested to promote inflammation and retinal cell death [9]. Multiple AMD-associated alleles have since been identified in complement genes and other pathways (as summarized in Section 3), solidifying a role of genetics in the pathogenesis of the disease. Environmental and lifestyle factors, such as smoking [10,11,12] and nutritional intake [11,13,14], are also known to contribute to the risk of AMD. A statistical modeling study by Seddon et al. estimated that environmental factors may contribute to 19–37% of AMD risk [12,15]. In addition, Seddon et al. used a monozygotic twin study to determine that AMD severity was increased in twins who smoked more heavily and had low dietary intake of vitamin D, compared with their co-twin, despite identical genetic backgrounds [12,15]. In particular, Millen et al. found that higher serum vitamin D levels were associated with reduced AMD risk, particularly among individuals with risk-conferring *CFH* and age-related maculopathy susceptibility 2 (*ARMS2*) genotypes [16]. Collectively, these findings suggest that both genetic and environmental risk factors are involved in modulating disease and give rise to the possibility that epigenetic mechanisms may help contextualize how these risk factors interact to influence AMD progression [17,18,19,20].

“Epigenetics” is defined as heritable changes in gene expression that occur without altering the DNA sequence [21]. Common epigenetic mechanisms include DNA methylation [22], histone modification [23], histone post-translational modifications [24], and non-coding RNA (ncRNA) [25]. These mechanisms work together to regulate chromatin structure and gene accessibility, thereby influencing transcription [26]. Supporting this, subsequent investigations have demonstrated associations between DNA methylation patterns and gene expression associated with oxidative stress, inflammation, and angiogenesis (summarized in Section 4.1). Moreover, alterations in histone acetylation have been linked to regulating inflammatory genes [17,27] (summarized in Section 4.2),. Unlike mutations, most epigenetic processes are reversible, therefore, therapeutic interventions for epigenetic-driven pathologies are possible, with some therapies approved for clinical use [28].

Epigenetic dysregulation has been extensively studied in other diseases, including cancer [29], metabolic disorders [30,31], neurodegenerative disease [32,33], and other age-related conditions [34,35]. This review aims to discuss recent advancements in genetic and epigenetic research, highlighting progress, remaining knowledge gaps in current research and outlining future directions for epigenetic research in AMD.

## 2. The Pathophysiology of Age-Related Macular Degeneration

**Figure 1:**
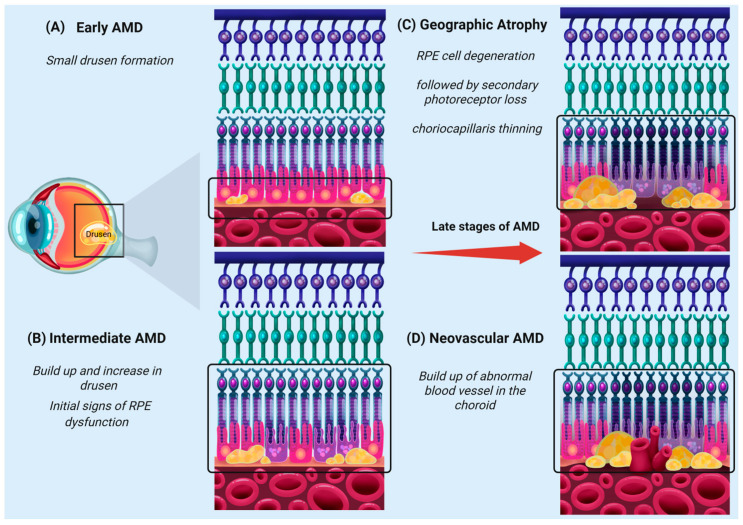
Stages of AMD progression. Key cellular and structural changes observed from early to late stages of disease. (**A**) Early AMD marked by the formation of small- to medium-sized drusen between the RPE and BrM. (**B**) Intermediate AMD is characterized by larger, confluent drusen formation as well as initial signs of RPE dysfunction. (**C**) Geographic atrophy (GA) is represented by RPE cell degeneration followed by secondary photoreceptor loss and choriocapillaris thinning. (**D**) Neovascular AMD (nAMD) is represented by abnormal blood vessel formation in the choroid through BrM into the RPE and retina, which causes leakage, hemorrhage, and scarring. This Figure was created using Procreate 5.3.15 and modified using Biorender [https://BioRender.com].

Although the precise pathophysiological mechanisms underlying AMD are not fully understood, numerous studies have examined potential contributing factors, as reviewed in [36,37]. A hallmark of AMD is the accumulation of yellow extracellular deposits known as drusen, which are composed of lipids, proteins, and cellular debris [38]. These build up between the RPE and BrM in early AMD [4,39], as illustrated in Figure 1. Clinically, disease progression is marked by an increase in the number and size of the drusen, as well as their distribution across the macula [38,40,41,42]. These changes are associated with molecular alterations, including inflammation and oxidative stress, which impair outer retinal function and contribute to photoreceptor loss [4,39]. The structural and biochemical changes in BrM precede RPE degeneration and play a role in early AMD pathogenesis [43]. With aging, the BrM undergoes progressive thickening, lipid accumulation, cross-linking of collagen and elastin fibers, which impair its permeability hindering nutrient, waste, and metabolite exchange between the choroid and RPE [43,44]. These alterations compromise the local microenvironment, contributing to oxidative stress and the accumulation of extracellular debris [38,43,44].

Dry AMD is estimated to comprise approximately 85–90% of AMD cases [45,46]. The advanced form of dry AMD, known as geographic atrophy (GA) or ‘atrophic AMD’, and is categorized by a widespread degeneration of RPE in the macula, accompanied by loss of adjacent photoreceptors and choriocapillaris [46,47,48,49]. GA lesions may expand over time, leading to progressive degeneration and vision loss [50], as illustrated in Figure 1, and patients with GA have reported challenges with everyday tasks and a decline in vision [51,52,53]. The precise indicators that give rise to the onset and progression of GA are still debated, though factors such as oxidative stress and inflammatory pathways, including the complement cascade, have been implicated [54,55].

In contrast, neovascular AMD (nAMD) makes up 10–15% of AMD cases [46,47,56,57]. It is characterized by the development of choroidal neovascularization (CNV), a pathological process where delicate blood vessels originating from the choroid diffuse into the RPE of the macula region [58,59]. These vessels are prone to leakage or hemorrhage into the outer retinal space, leading to the disruption of photoreceptor structure and function [58,59] (Figure 1)**.** Clinically, patients presenting with nAMD report acute visual disturbance, increased central blur, and displacement of straight lines [60]. The pathogenesis of nAMD is thought to involve a range of factors, including the dysregulation of vascular endothelial growth factor A (VEGFA), as well as oxidative damage, and inflammatory pathways such as the complement cascade [59,61,62].

Treatment options vary depending on AMD subtype. Anti-VEGF therapies have transformed the treatment of nAMD by lowering macular edema and repressing CNV [63,64,65]. Clinical trials, including Anti-VEGF Antibody for the Treatment of Predominantly Classic Choroidal Neovascularization in Age-Related Macular Degeneration (ANCHOR) [66], Minimally Classic/Occult Trial of the Anti-VEGF Antibody Ranibizumab in the Treatment of Neovascular Age-Related Macular Degeneration MARINA [66], and the OAKS [67] and DERBY [67] trials, have demonstrated that these therapies effectively preserve and improve central vision. Rather than relying on fixed monthly or bi-monthly dosing, many clinics have now adopted a “treat-and-extend” strategy, where dosing intervals are adjusted based on disease activity [68]. This approach has been shown to maintain visual outcomes while reducing treatment frequency [68].

Historically, there have been no approved treatments for slowing or preventing the progression of GA. Recent advances, however, have led to the approval of complement inhibitors that slow the progression of GA lesions. Syfovre (pegcetacoplan), a complement 3 inhibitor (*C3*), has been shown in trials to significantly slow the progression of GA lesion size [67]. Though a promising breakthrough in GA management, the widespread utility of Syfovre is currently debated due to reported side effects, such as CNV [69,70]. Izervay (avancincapted pegol), targeting complement 5 (*C5*), has also received recent approval for GA as of 2024 and was found in previous trials to yield a 27–30% reduction in GA lesion size [71,72,73].

## 3. Genetic Risk Factors in Age-Related Macular Degeneration

**Figure 2:**
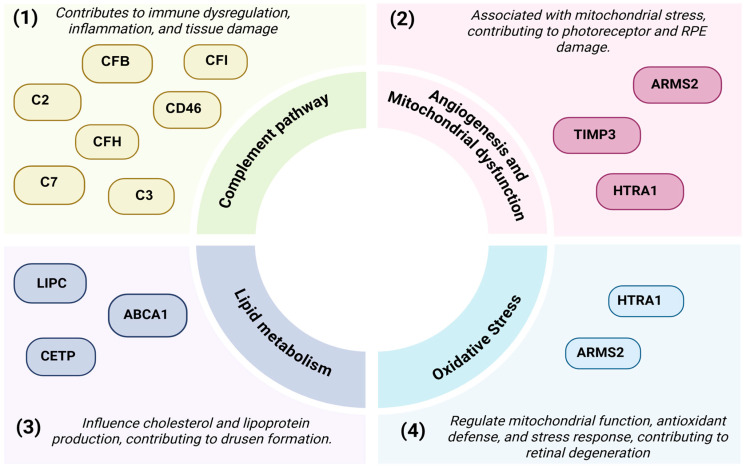
Genetic risk factors associated with AMD pathogenesis. Key genes associated with AMD pathogenesis classified into four subtypes: (**1**) the complement pathway genes, which may contribute to immune dysregulation, inflammation, and degeneration of the retina. (**2**) Angiogenesis and mitochondrial dysfunction genes, involved in CNV development, mitochondrial dysregulation, RPE degeneration, and photoreceptor loss. (**3**) Genes involved in lipid metabolism, influencing cholesterol and lipoprotein production, influencing drusen formation. (**4**) Genes associated with enhancing oxidative stress, influencing the regulation of mitochondria, immune cells, and stress responses, promoting retinal degeneration. This image was created using Biorender [https://BioRender.com] and Procreate.

AMD is recognized as a complex polygenic disorder, influenced by numerous genes and single-nucleotide polymorphisms (SNPs) [74]. In fact, genetic factors are estimated to account for 47–71% of AMD cases [12]. Research involving family history, twin studies, and segregation analyses strongly supports the heritability of AMD, providing consistent evidence that genetic factors significantly contribute to disease risk [12,74,75,76]. The mapping of susceptibility loci in AMD was initially accomplished by family-based linkage studies [77,78]. For instance, a link to chromosome 1q25-31 was found in an AMD pedigree with autosomal dominant GA [77], a finding that was confirmed by other studies [79,80]. AMD genetics has been comprehensively reviewed in [81,82], outlining the major biological pathways associated with disease progression, including inflammation, lipid metabolism, oxidative stress, and angiogenesis. Genes discussed in this review have been summarized in Figure 2.

GWASs have significantly advanced the understanding of the genetic architecture of AMD. The first major discovery was published in 2005, which suggested that the complement pathway is involved in AMD development, with multiple risk variants identified within *CFH* [7]. Other large-scale studies have expanded on these findings. For instance, Fritsche et al. identified 34 gene loci associated with increased AMD risk [83], while subsequent studies identified over 100 additional genetic variants associated with AMD [76,84,85].

One of the most well-characterized polymorphisms is Tyr402His (Y402H), a single amino acid change in the *CFH* domain [86,87]. It has been demonstrated that the Y402H variant has a lower binding affinity with ligands, especially the heparan sulfate found in the BrM [88]. As a result, the Y402H variant suppresses complement activation less effectively, contributing to dysregulation of the complement pathway [6,7,89,90]. Research has suggested that individuals who carry the Y402H mutation have a significantly increased risk of developing AMD compared to the control group [90]. Other genes in the complement cascade have also been associated with AMD; these are illustrated in Figure 2. For instance, studies have investigated *C3* [76,91], complement component 2 (*C2*) [92], complement Factor B (*CFB*) [93], complement 7 (*C7*) [94], cluster of differentiation 46 (*CD46*) [95], and complement factor I (*CFI*) [96,97]. The findings above suggest that genes linked to complement dysregulation and inflammation are important contributors to the pathophysiology of AMD.

Studies have also highlighted an association between two tightly linked genes, *ARMS2* [98,99] and the adjacent high-temperature requirement A serine peptidase 1 (*HTRA1*) [98,100], both located on chromosome 10q26. *ARMS2* is thought to encode a mitochondria-associated transcript, most likely functioning as a long noncoding RNA involved in modulating oxidative stress and mitochondrial homeostasis in the RPE [89,98,101,102]. In contrast, HTRA1 encodes a secreted serine protease that is involved in extracellular matrix (ECM) remodeling, though its exact functional consequence remains debated [98,100]. Studies have indicated that AMD-associated variants, including the rs11200638 polymorphism in the *HTRA1* promoter, lead to overexpression of *HTRA1* in RPE cells [98,100,103]. This is thought to promote ECM degradation and angiogenesis and may contribute to nAMD. Other studies have provided an alternative mechanism, where a haplotype associated with increased disease susceptibility may impair expression of an RNA element within the *ARMS2* exon1-intron1 region, resulting in reduced *HTRA1* levels [104]. These contrasting findings imply a complex interaction at the *ARMS2*/*HTRA1* locus and indicate a need for further investigation.

Rare mutations in the tissue inhibitor of metalloproteinases 3 gene (*TIMP3*), a key regulator of the ECM [105], have been linked to nAMD via a comprehensive GWAS analysis [83], which revealed potential associations with other ECM-related genes, such as matrix metalloproteinase-9 (*MMP9*). In addition, several genetic risk factors related to lipid metabolism and oxidative stress pathways have also been identified as contributing to AMD pathogenesis [76,106,107,108]. Genes including hepatic lipase (*LIPC*) [109,110], cholesteryl ester transfer protein (*CETP*) [109], and ATP-binding cassette transporter A1 (*ABCA1*) [111,112] influence the production of cholesterol and lipoprotein, associated with drusen formation [112] (Figure 2). A potential link has also been suggested between a splice variant of the retinal G protein-coupled receptor (*RGR*) gene and the development of AMD-like characteristics [113,114]. This variant results from an alternative splicing event skipping exon 6, known as RGR-delta-6 (*RGR*-d), which lacks a transmembrane domain. The accumulation of RGR-d is associated with drusen deposits and has been shown to elicit ‘dry’ AMD-like pathology in RPE cells in vitro, and in aged and high-fat diet experimental mice [113,114,115].

The genetic variants associated with AMD are complex and are influenced by a variety of factors. Researchers have developed polygenic risk scores (PRS) [116] to determine the risk of numerous susceptibility variations associated with AMD patients. This could potentially provide personalized genetic treatment for patients and early disease detection [116].

## 4. Evidence for Epigenetic Regulation in Age-Related Macular Degeneration

**Figure 3:**
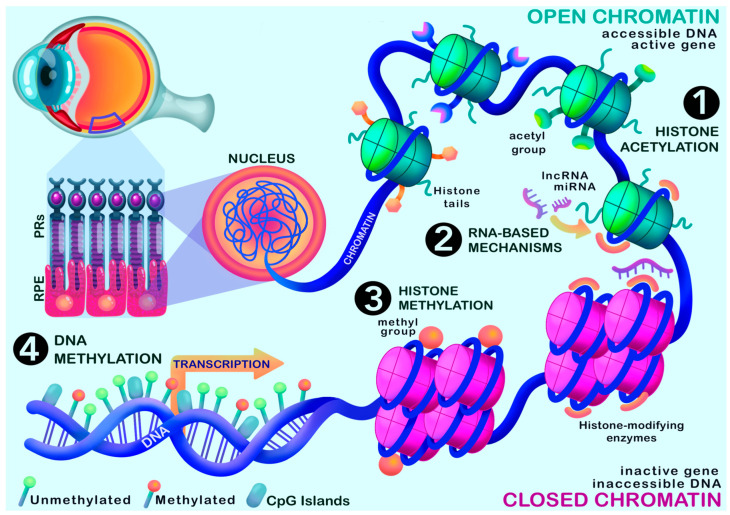
Epigenetic mechanisms that have been investigated in the context of AMD. (**1**) Histone acetylation. (**2**) RNA-based mechanisms, including long noncoding RNA (lncRNA) and micro-RNA (miRNA). (**3**) Histone methylation. (**4**) DNA methylation. Mechanisms that promote open chromatin are signified by green histones, whereas mechanisms that promote closed chromatin are signified by pink histones. This image was created using Procreate.

Epigenetics has become an area of significant interest in many diseases, including AMD [117,118,119]. Epigenetic switches have been shown to act as moderators influencing disease onset and progression, such as DNA methylation [22], post-translational modifications (PTMs) of histone proteins [120], 3D chromatin structure [121], nucleosome positioning [122], and regulatory actions of non-coding RNAs (ncRNA) [25], as illustrated in Figure 3. Together, these modifications work by regulating the expression of genes, influencing chromatin structure and DNA accessibility [17].

### 4.1. DNA Methylation

DNA methylation, a key epigenetic modification, involves the covalent binding of a methyl group to the C5 position of cytosine within CpG dinucleotides, forming 5-methyl cytosine [123,124,125] (Figure 3). When occurring at gene promoters, this modification is typically linked to transcriptional repression [123,124,125]. The retina is highly susceptible to oxidative damage due to its high metabolic activity. Hence, methylation patterns are involved in maintaining gene expression homeostasis [126,127].

**Figure 4:**
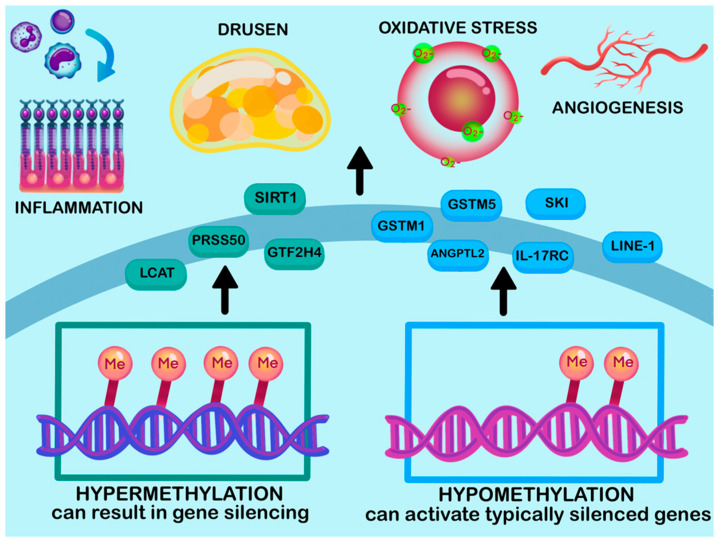
Proposed interactions between epigenetic-genetic factors influencing AMD progression pathways. Epigenetic changes in methyl groups attached to cytosines in CpG dinucleotides (Me) act as epigenetic switches in genes associated with AMD onset and progression. Processes involved in AMD progression, inflammation, drusen formation, oxidative stress, and angiogenesis are driven by contrasting methylation patterns in a number of genes. Hypermethylation of promoter regions, where increased methyl groups are added to DNA, results in changes of gene expression in *SIRT1* [132], *LCAT* [139], *PRSS50* [136], and *GTF2H4* [137] (**left**), which then drive AMD processes. In contrast, hypomethylated promoter regions, with decreased methylated bases, in *GSTM1*, *GSTM5* [129,130], *SKI* [129,137], *ANGPTL2* [138,145], *IL17RC* [131,144], and *LINE-1* [132,139,140,141] (**right**) are associated with these AMD processes. This image was created using Procreate.

Evidence indicates that aberrant DNA methylation affects key disease-related pathways, such as oxidative stress, immune regulation, and mitochondrial function (Table 1) [128]. For example, using quantitative trait locus mapping (QTLM) on human retinal tissues, Advani et al. found 87 genes whose methylation status was substantially associated with an elevated risk of AMD [129]. Both Advani et al. [129] and earlier work by Hunter et al. [130] identified hypomethylation in glutathione S-transferase isoform mu1 (*GSTM1*) and mu5 (*GSTM5*) in AMD tissues, associated with a reduction in mRNA expression, antioxidant defenses, and increased retinal damage [129,130] (Figure 4, Table 1).

Other studies elucidate the key function of methylation patterns in oxidative stress response by examining DNA methyltransferase (DNMT), an essential enzyme in charge of preserving the DNA methylation patterns already present in cells [131]. In contrast, findings by Maugeri et al. revealed that, in the early stages of AMD, DNMT activity increased by 48% [132]. This hypermethylation resulted in silencing of protective gene promoters such as *SIRT1*, resulting in a loss of cellular function, increased vulnerability to oxidative stress, inflammation, and tissue damage, which may result in disease progression [132]. These findings highlight the importance of analyzing methylation dynamics across disease stages and within specific retinal cell types.

In addition, DNA methylation may potentially affect the inflammatory pathways associated with AMD. Wei et al. [133] and Wang et al. [134] demonstrated hypomethylation and overexpression of interleukin 17 receptor C (*IL17RC*) in AMD. However, opposing results were communicated by Oliver et al. [135], indicating no significant difference in the methylation of *IL17RC* in the blood of AMD patients, and that serine protease 50 (*PRSS50*), a protein-encoding gene, exhibited increased DNA methylation inpromoter regions, promoting proliferation [136]. The variability in results underscores the need for tissue-specific studies, as both gene targets and methodological differences may influence observed methylation changes in AMD.

Similarly, hypomethylation of the *SKI* [129,137] and angiopoietin-like 2 (*ANGPTL2*) [138] have been linked to dysregulation of inflammation, fibrosis, and enhancement of angiogenesis, which contributes to the development of nAMD. In contrast, general transcription factor IIH subunit 4 (*GTF2H4*) showed hypermethylation, suggesting a potential role in impairing genomic integrity in AMD tissues, as this gene is usually responsible for DNA repair [137].

Environmental risk factors may also be associated with epigenetic mechanisms in AMD. A recent study investigated DNA methylation of the lecithin–cholesterol acyltransferase (*LCAT*) gene, which plays a role in lipid metabolism, and identified an association with increased AMD risk [139]. Hypermethylation of the *LCAT* promoter was associated with reduced gene expression. This indicates that inadequate antioxidant consumption may epigenetically inhibit lipid-regulating genes, facilitating the formation of drusen and causing retinal degeneration as a result, increasing susceptibility to disease [139]. Simultaneously, AMD patients showed global hypomethylation of long interspersed nuclear element *(LINE)-1*, suggesting its potential involvement in DNA damage, replication stress, and genomic instability, which can contribute to disease progression [132,139,140,141].

In summary, localized changes in DNA methylation patterns (either hypermethylation or hypomethylation) interfere with the homeostatic expression of genes, contributing to AMD pathogenic mechanisms, including inflammation, oxidative stress, and angiogenesis [119]. Further investigation of DNA methylation patterns may provide potential for discovering early biomarkers of disease, which may facilitate early detection and accurate diagnosis. Additionally, studies suggest that discrete retinal cell populations may exhibit distinct DNA methylation patterns [142]. Considering that the retina comprises a complex multicellular architecture, it would also be advantageous to use single-cell methylation profiling [143] to investigate cell-specific DNA methylation patterns. This approach may enhance our understanding of how the disease progresses in response to stressors, such as oxidative stress and inflammation, and provide data on the changes associated with early and advanced AMD.

**Table 1:** Overview of DNA methylation patterns and their potential link to AMD.

Name of Gene	Methylation Status	Tissue or Source	Regulation in AMD	Proposed Function	Reference
*GSTM1* and *GSTM5*	Hypomethylated	RPE/choroid	Downregulated	Reduces antioxidant defense. Involved in increasing RPE vulnerability Involved in oxidative stress response	[129,130]
*IL17RC*	Hypomethylated	Blood and Retina	Upregulated	Enhances chronic inflammation	[131,144]
*ANGPTL2*	Hypomethylated	AMD retina	Upregulated	Involved in Angiogenesis Increases risk of CNV	[138,145]
*SKI*	Hypomethylated	AMD RPE	Upregulated	Impacts oxidative stress pathway Associated with TGF-β signaling	[129,137]
*GTF2H4*	Hypermethylated	AMD RPE	Downregulated	Impaired DNA repair and transcription, affecting degeneration	[137]
*LINE-1*	Hypomethylated	Peripheral Blood	Downregulated	Increased transcription and genomic instability	[132,139,140,141]

Abbreviations: *ANGPTL2*: angiopoietin-like protein 2, *GTF2H4*: general transcription factor IIH subunit 4, *GSTM1*: glutathione S-transferase isoform mu1, *GSTM5*: glutathione S-transferase isoform mu5, *IL17RC*: interleukin 17 receptor C, *LINE-1*: long interspersed nuclear element, long interspersed nuclear element-1, *SKI*: SKI proto-oncogene.

### 4.2. Histone Variants and Modifiers

The basic unit of chromatin is the nucleosome, comprising 147 base pairs of DNA wrapped around an octamer of core histones [146,147,148,149]. The histone octamer consists of a central H3-H4 heterotetramer flanked by H2A and H2B dimers [146,147,148]. Histone variants can substitute core histones in nucleosomes, conferring distinct regulatory functions related to transcription, DNA repair [23,148,149], and chromatin structure [149]. Histone modifications, such as acetylation and methylation, modulate gene accessibility and transcriptional activity [147,150]. Histone acetylation [151,152] reduces DNA–histone binding, leading to an open chromatin structure and promoting transcription [151,152]. In contrast, histone methylation can either activate or repress transcription, depending on the specific modification site [153,154].

To date, one study by Dubey et al. [27] has examined age-related histone changes in the RPE compared to the retina in mice [27]. Results demonstrated a global reduction in the linker histone H1, as well as the core histones H2A, H2B, H3, and H4 in the RPE/choroid by 40–55% in aged mice compared to young mice [27]. The histone loss was specific to the RPE, while the neural retina maintained the same amount of histone expression between aged and young mice [27]. Furthermore, the acetylation of H3K14, H3K56, and H4K16 was reduced in aged RPE/choroid cells, suggesting a shift in the balance of histone acetyltransferase (HAT) and histone deacetylase (HDAC) activity [27]. This imbalance may contribute to altered chromatin compaction and gene expression [27]. While this study provides evidence of histone loss and reduced acetylation during RPE aging, the question of its direct applicability to AMD remains to be elucidated.

Dubey et al. also reported a global reduction in the histone H3 levels of aged RPE cells. The contribution of the replication-independent variant H3.3 remains unclear, as the study did not perform a variant-specific analysis. This warrants further investigation, as H3.3 is expected to accumulate with age, as it replaces the canonical histones H3.1 and H3.2 during the S-phase [27]. Future research should include in vivo AMD models to track H3.3 levels or employ single-cell chromatin-immunoprecipitation sequencing (ChIP-seq) [155] to determine the cell-type-specific regulation and chromatin patterns in the disease state.

**Figure 5:**
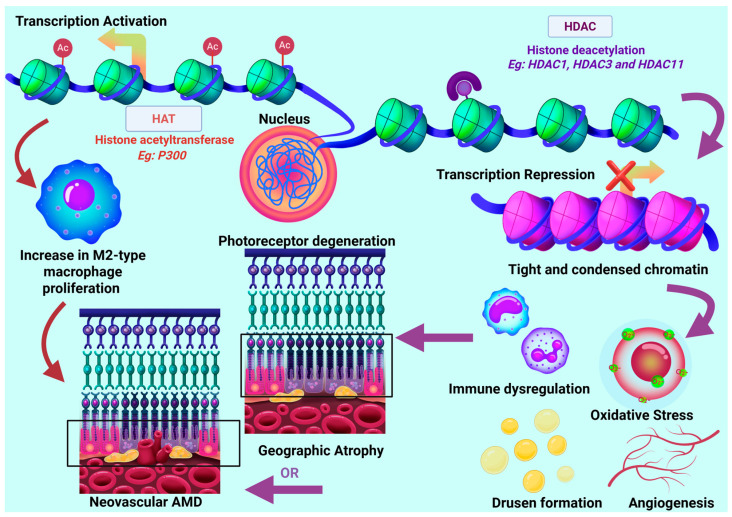
Influence of HDAC and HATs in AMD pathogenesis. Histone acetylation (HATs), such as P300, function by supporting an open chromatin state, activating transcription, which may result in increasing M2-type macrophage proliferation associated with increased susceptibility to nAMD [4,174]. Whereas, histone deacetylases (HDACs) support a closed chromatin state (condensation), inhibiting transcription [156]. Studies have demonstrated downregulation of *HDAC1* [157,159] and *HDAC3* [157,158] and downregulation of *HDAC11* [160,177,178]. This causes dysregulation of key cellular processes, increasing susceptibility to inflammation, immunological dysregulation, oxidative stress, and angiogenesis, resulting in either nAMD or GA [19,157,158,159,160]. This Figure was created using Procreate and modified using Biorender [https://BioRender.com].

In addition to histone loss, the altered expression of HDACs has been linked to AMD pathophysiology (see Table 2). HDACs remove acetyl groups from histones, modulating chromatin compaction and influencing transcriptional accessibility [156]. Studies using mouse models have demonstrated a downregulation of *HDAC1* [157] in GA, suggesting a role in promoting inflammation [27,157] and *HDAC3* [157,158]. These HDACs may support the transcription associated with oxidative stress and immune regulation pathways [19,157,158,159,160]. In contrast, elevated levels of *HDAC11* expression were reported in GA [161,162], and its involvement in RPE dysfunction, chromatin compaction, and inflammatory responses has been suggested [161,162]. Figure 5 illustrates that AMD pathogenesis pathways, such as inflammation, oxidative stress, and angiogenesis, may be influenced by HDAC dysregulation. However, the studies discussed above are based on mouse models and require validation in human retinal tissues.

Conversely, the findings from AMD cybrid models have revealed inconsistent patterns of expression between HDAC genes, reflecting the complexity of epigenetic regulation in disease states (see Table 2) [19]. In these models, *HDAC1*, *HDAC2*, and *HDAC3* expression was elevated, while *HDAC6*, *HDAC9*, and *HDAC10* were downregulated [19]. These differential patterns highlight the complicated relationship between mitochondrial dysfunction and HDAC regulation in AMD. Cybrid models are limited by mitochondrial–nuclear cross compatibility and cell line mutations. As a result, investigations need to be confirmed in humans, ideally with longitudinal studies to characterize the HDAC expression at different stages of AMD.

Furthermore, it has also been suggested that *SIRT1* plays a role in maintaining retinal homeostasis [163]. *SIRT1* activation by resveratrol has been shown to downregulate VEGF and hypoxia-inducible factor 1-alpha (*HIF1A*), reducing oxidative stress and angiogenesis [132,164,165,166]. Additional research has implied that *SIRT1* may have protective functions against inflammation [164,167,168,169,170] and neurodegenerative diseases [171,172,173], supporting its role as a potential therapeutic target. P300 histone acetyltransferase (HAT) has also been found to improve the stability of X-box binding protein 1 (XBP1s) and increase the transcriptional activity of its target, homocysteine inducible endoplasmic reticulum protein with ubiquitin-like domain 1 (*Herpud1*), which promotes the polarization of M2 macrophages. The inhibition of this axis in cultured RAW264.7 cells reduced the migration and proliferation of mouse choroidal endothelial cells in culture. Further in vivo experiments in a laser-induced mouse model of CNV confirmed that the inhibition of this axis reduced the polarization of M2 macrophages and the development of CNV lesions [174].

Together, these investigations suggest that histone variant loss and dysregulated HDAC and HAT activity may contribute to AMD onset and progression (summarized in Figure 5). However, more investigation is needed to establish whether changes in HDAC and HAT expressions are key causative features or subsequent reactions to cellular stressors such as oxidative stress and inflammation. Given that histone modifications are dynamic and reversible, they present promising therapeutic targets. While research on HDAC in AMD is expanding, studies on HATs remain limited. Exploring their role may improve understanding of disease progression, especially as some evidence suggests that HATs are involved in photoreceptor degeneration and differentiation [175]. Finally, there remains a gap in our understanding of the interplay between histone modification, DNA methylation, and chromatin accessibility. Single-cell epigenomic techniques, including ChIP-seq [155] and ATAC-seq [176], may offer insights into cell-specific epigenetic changes during AMD development.

**Table 2:** Detailed overview of HDAC’s potential role in AMD.

HDAC Isoform	Expression Changes	Proposed Role/Function	Reference
HDAC1 and HDAC2	Downregulated in retinal cells with advanced GA. Upregulated in cybrid model	Chromatin compaction transcription repression DNA damage response. Represses Inflammation	[19,27,157]
HDAC 3	Downregulated in retinal models Upregulated in cybrid models	Modulates oxidative stress and immune response	[19,157,158]
HDAC9	Downregulated in cybrid AMD models	Regulation of angiogenesis, apoptosis, inflammation	[19]
HDAC10	Downregulated in cybrid AMD models	Regulation of metabolic and cellular stress response.	[19]
HDAC11	Upregulated in retinal and cybrid AMD models	Regulates Inflammation. Prompts photoreceptor degeneration.	[19,161,162]
SIRT1	Downregulated in retinal AMD models	Regulates expression of VEGF. Protect against oxidative stress Regulates inflammation	[132,164,165,166]

Abbreviations: GA: geographic atrophy, HDAC: histone deacetylase.

### 4.3. Chromatin Accessibility

Epigenetic modifications shape chromatin conformation and influence the genome’s three-dimensional architecture in both health and disease [179]. Chromatin exists in an open configuration that enables gene transcription or a compacted state that restricts access to regulatory elements [180,181]. Using ATAC-seq [182], decreases in chromatin accessibility have been observed in AMD [161,162], alongside decreased H3K27ac levels in the euchromatin region and increased H3K9me3 in the heterochromatin region of RPE/choroid tissues [161,162]. These findings suggest that early epigenetic changes in RPE cells may contribute to AMD by disrupting the regulatory pathways linked to inflammation, oxidative stress, and angiogenesis. Supporting this, Smith et al. [183] developed an integrated epigenomic and transcriptome map of induced pluripotent stem cells in the RPE and combined them with adult retina/RPE cells [183]. The study identified rs943080, a non-coding SNP at the *VEGFA* locus, as a risk allele that may reduce *VEGFA* expression and chromatin accessibility in the retina [183]. This study demonstrates how genetic variations can alter gene regulation and contribute to AMD pathogenic pathways by altering chromatin accessibility. For instance, reduced chromatin accessibility at angiogenesis-associated loci like *VEGFA* may impair vascular regulation and contribute to CNV, a hallmark of nAMD. Single-cell ATAC-seq (scATAC-seq) [184] could further resolve chromatin states for individual cell types.

While the aforementioned studies have served to broaden our understanding of chromatin accessibility changes in AMD, there remain unanswered questions. Post-translational modifications (PTMs) that control enhancer and repressive chromatin states, such as H3K27me3 [185] and H3K4me1 [186], have not been well-characterized in AMD and could reveal early epigenetic indicators of disease development and pathophysiology. Additionally, it is unclear how nuclear envelope permeability and environmental risk factors, such as smoking, may interact with the epigenome. Integrating ATAC-seq with DNA methylation profiles could enhance our understanding of gene regulation in AMD and uncover disease-specific epigenetic profiles. Such studies should consider applying multi-omic tools, including single-nucleus chromatin accessibility and mRNA expression sequencing (SNARE-seq) [187] and simultaneous high-throughput ATAC and RNA expression with sequencing (SHARE-seq) [188] in AMD-based models.

### 4.4. Long Noncoding RNA

Non-coding RNAs (ncRNAs) are functional molecules that do not encode proteins but regulate gene expression by modulating transcriptional activity [189,190]. NcRNAs may also operate as epigenetic regulators through targeting chromatin alterations or influencing transcriptional levels. NcRNAs are typically categorized by their length: short ncRNAs are less than 200 nucleotides [191], while long non-coding RNAs (lncRNAs) exceed 200 nucleotides in length [192]. Small interfering RNAs (siRNAs) [193,194] and microRNAs (miRNAs) [195,196] have also been well-researched in the context of AMD and other retinal diseases (as reviewed in [194,197]). However, the scope of this review will focus on emerging lines of evidence for a potential link between lncRNA dysregulation and the pathogenesis of AMD. Microarray/RNAseq analyses in both AMD patient tissues and experimental models have yielded dozens of potential lncRNA candidates that are disease-associated, and a number of lncRNA candidates that are particularly well-characterized are summarized in Table 3.

Several studies have suggested a possible role of lncRNAs in modulating angiogenic and immunological pathways in AMD. The lncRNA known as VAX2 homeobox transcription factor gene transcribed from opposite side (*VAX2OS1*) was among the first identified to regulate retinal development by modulating cell-cycle progression [198]. *VAX2OS1* and *VAX2OS2* are significantly upregulated in the aqueous humor of nAMD patients [198,199], suggesting their presence as a potential biomarker of neovascularization. *VAX2OS1* is predicted to interact with nuclear factor kappa B (NF-κB), which is linked to the elicitation of pro-inflammatory and pro-angiogenic pathways in AMD [200]. A study by Zhang and colleagues utilized a microarray analysis in a mouse experimental model of CNV, through which they identified 129 differentially expressed lncRNAs in CNV [201]. The authors narrowed their focus on the lncRNA H19 imprinted maternally expressed transcript (*H19*), where they indicated that suppression decreases markers for M2 macrophages in laser-induced CNV, suggesting a potential role for *H19* in mediating macrophage polarization. Finally, the study indicated that *H19* was elevated in the aqueous humor of CNV patients. Investigations have also alluded to a role for the lncRNA metastasis-associated lung adenocarcinoma transcript 1 (*MALAT1*) in CNV [202]. *MALAT1* was upregulated in choroidal tissues in experimental CNV in mice, while experimental knockdown decreased CNV lesion size. The authors further indicated that *MALAT1* may exert this effect by influencing the miR-17-5p, VEGF, and E26 transformation-specific-1 (ETS1) axis, using an in vitro model comprising human choroidal vascular endothelial cells (HCVECs) [202].

Other studies have indicated an association of lncRNAs with the dysfunction, degeneration, or dedifferentiation of RPE cells, features of which are linked to their pathogenesis in AMD [203]. For instance, Chen and colleagues illustrated that the long intergenic non-protein coding RNA 167 (*LINC00167*) was downregulated in macular RPE-choroid tissues from AMD patients [204,205]. The authors showed that silencing *LINC00167* in cultured RPE cells induced their differentiation, via an miR-203a-3p/SOCS3-mediated axis, indicating a role for *LINC00167* in promoting RPE function. The expression of lncRNA Prader–Willi region non-protein coding RNA 2 (*PWRN2*) has also been examined in cultured RPE cells, which were exposed to t-BuOOH-induced mitochondrial stress [206]. The upregulation of *PWRN2* in this study was found to be associated with aggravated apoptosis in RPE cells and mitochondrial damage.

In another study, Zhu and colleagues identified a total of 64 lncRNA candidates that were differentially expressed in RPE/choroid samples from early AMD patients by utilizing microarray data [207]. Of these, the lncRNA *RP11-234O6.2* was downregulated in early AMD, which the authors then selected for further investigation with an oxidative stress-induced RPE culture model. The study showed that *RP11-234O6.2* was likewise decreased in the RPE culture model, while transfection of *RP11-234O6.2* preserved RPE viability in response to oxidative stress. The precise role of *RP11-234O6.2* is unclear, though it has been predicted to interact with rod outer-segment membrane protein 1 (ROM1) mRNA [207]. Similarly, another investigation identified a downregulation of the lncRNA ZNF503 antisense RNA 1 (*ZNF5030-AS1*) in the RPE/choroid of AMD patients [208]. *ZNF5030-AS1* was associated with promoting RPE differentiation in the same study, using a human induced pluripotent stem cell (hiPSC) RPE culture model. Conversely, the silencing of *ZNF5030-AS1* was found to induce RPE dedifferentiation [208].

Other candidate lnRNAs identified in recent studies include maternally expressed gene 3 (*MEG3*) and a potential link to photoreceptor apoptosis. Zhu and colleagues showed that *MEG3* is upregulated in photooxidative damage in mice, used to model features of dry AMD, while experimental suppression of *MEG3* ameliorated photoreceptor cell death in photooxidative damage and in light-stressed 661W in vitro [209]. Additionally, a GWAS identified a novel variant within the lncRNA region known as *AC103876.1* near Parkinsonism-associated deglycase (PARK7) and Teneurin-3 transmembrane protein 3 (TENM3), which was significantly associated with AMD, despite being located outside of known AMD risk gene loci. However, the exact functional role of *AC103876.1* remains to be elucidated [210].

Overall, these studies highlight the potential role of lncRNA in the pathogenesis of AMD, as summarized in Table 3. LncRNA candidates continue to be characterized, as shown by the recent identification of a novel lncRNA *BX842242.1*, located antisense and upstream of *HTRA1*, which is associated with an increased risk of reticular pseudodrusen in AMD [211]. While promising, it must be noted that this particular study has not yet completed peer review.

**Table 3:** Overview of lncRNA candidates that have been linked to AMD pathogenesis.

Name of lncRNA	Expression Changes	Proposed Role	Tissue/Model	Reference
*RP11-234O6.2*	Downregulated	Downregulated in RPE/choroid of AMD patients, implicated in protecting RPE cells from oxidative damage-induced cell death.	AMD RPE/choroid Human RPE culture model	[207]
*PWRN2*	Upregulated	Involved in promoting RPE cell death and stress-related mitochondrial damage.	Human RPE culture model	[206]
*MEG3*	Upregulated	Implicated in promoting apoptosis via association with p53 transactivation.	Mouse photooxidative damage model	[209]
*Vax2os1*, *Vax2os2*	Upregulated	Enriched in aqueous humor of nAMD patients. Predicted interaction with NFκB, involved in inflammation and angiogenesis.	nAMD aqueous humor	[198,199]
*MALAT1*	Upregulated	Increased in experimental CNV, suppression reduces CNV lesion size. Implicated in promoting choroidal neovascularisation via modulation of VEGF-A expression.	Mouse CNV model	[212]
*ZNF503-AS1*	Downregulated	Decreased in RPE/choroid of GA patient specimens. Implicated RPE protection by suppressing dedifferentiation and pathology, in vitro.	AMD RPE/choroid Human RPE culture model	[208,213]
*LINC00167*	Downregulated	Decreased in RPE/choroid of AMD patient specimens. Suppression promotes RPE dedifferentiation and mitochondrial/phagocytic dysfunction in vitro, indicative of a protective role.	AMD RPE/choroid Human RPE culture model	[204,205]

Abbreviations: *LINC00167*, long intergenic non-protein-coding RNA 167; *MALAT1*, metastasis-associated lung adenocarcinoma transcript 1; *MEG3*, maternally expressed gene 3; *PWRN2*, Prader–Willi region non-protein coding RNA 2; *RP11-234O6.2*, long noncoding; *Vax2os1*/*Vax2os2*, VAX2 opposite strand transcript 1 and 2; *ZNF503-AS1*, zinc finger protein 503 antisense RNA 1.

Although these lncRNA studies to date have yielded promising links with AMD, it should be noted that the mechanistic underpinnings of many lean heavily on RPE monoculture experiments. Further validation, either in vivo or in complex culture systems, could offer valuable mechanistic insight into their intersection with AMD pathophysiology. Moreover, future studies could benefit from integrating lncRNA data with epigenomic and transcriptomic profiles for validating their association with AMD. This may potentially result in revealing novel biomarkers and therapeutic targets related to lncRNA and AMD.

## 5. Epigenetics as a Potential Therapeutic Target?

Though the advent of anti-VEGF therapies has undoubtedly improved the management of nAMD, challenges remain due to variable patient responses and a lack of treatments for the atrophic form of the disease [214]. Epigenetic regulation plays an essential role in gene expression and, with further investigation, may offer a potential therapeutic approach for complex degenerative diseases, including AMD [17,20,175,215].

Emerging evidence has linked DNA methylation patterns to AMD pathogenesis [130,137,216,217], as summarized in Table 1. For example, *DNMT1* is downregulated in the late stages of AMD compared to the early stages, and thus, it may serve as a potential biomarker of disease progression [132,216]. Studies have investigated the inhibition of 5-aza-2′-deoxycytidine (5-AZA-dc), a hypomethylating agent known to suppress angiogenesis and upregulate clusterin (*CLU*) expression in RPE cells [130]. This mechanism has been widely used in cancer treatments [218], but requires further investigation in ocular disease. Future investigations should explore DNMT inhibitors in different disease states (early, GA, and nAMD) to study how they may influence disease pathogenesis, onset, and progression. In addition, the effect of DNA methylation patterns on VEGFA expression should be explored to identify potential therapeutic targets. Moreover, DNA methylation is generally more stable in differentiated cells, such as the RPE, which presents a challenge for translational epigenetic therapies in AMD.

Furthermore, histone deacetylase inhibitors (HDACi) have been linked to regulating inflammation, oxidative stress, and aging, crucial to AMD pathogenesis or other retinal disorders [19,157,159] (Figure 4). For instance, in mouse models of retinitis pigmentosa (RP), HDACi trichoastin A (TSA) injections reduced cone photoreceptor cell death [219,220,221]. Other studies confirmed this finding by investigating TSA inhibition on *HDAC6*, which protected photoreceptors from oxidative damage by increasing the expression of chaperones involved in protein stability and cell survival [19,160,222]. Additional findings show that TSA inhibition reduces oxidative stress in RPE cells when exposed to hydrogen peroxide [223,224].

The overexpression of *HDAC11* has been shown to reduce chromatin accessibility, contributing to transcriptional repression in retinal tissues. Thus, the inhibition of *HDAC11* [19,161,225] has been linked to protection against ischemic retinal damage and photoreceptor degeneration [19,161,177,225]. Furthermore, inhibiting *HDAC1/2* with romidepsin caused a disorganization of junctions in human RPE cells [226,227] accompanied by an increase in acetylation of H3 and H4 histone marks [226,227]. This indicates that HDAC inhibition may influence histone levels [226,227]. Future research exploring histone-specific targets, along with HDACi, could offer a more targeted therapeutic approach.

**Figure 6:**
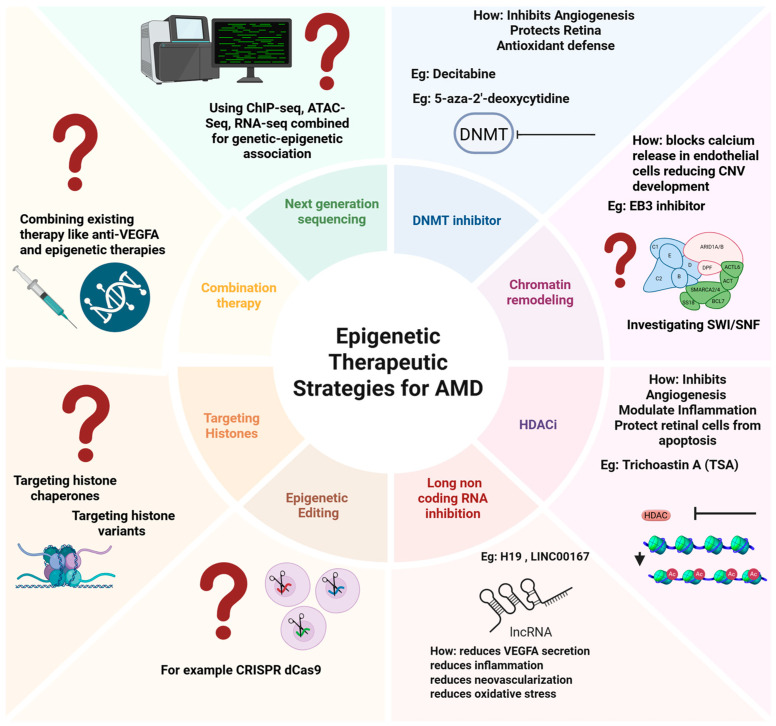
Potential epigenetic therapeutic candidates in relation to AMD, and suggestions for future directions. Previously established targets for epigenetic mechanisms and suggested further directions, including: Constructing genome-wide epigenomic maps to identify enhancers, silencers, and super-enhancers; Histone variants (e.g., H3.3) and chaperones regulating cell survival; CRISPR-dCas9 tools to modulate DNA methylation and histone acetylation in disease; Next-generation sequencing (ChIP-seq, ATAC-seq, and RNA-seq) to identify transcription factors and chromatin states. This image has been created using Biorender [https://BioRender.com].

End-binding protein 3 (EB3) is a chromatin remodeling factor [228,229], the inhibition of which has been shown to reduce neovascularization in laser-induced CNV mouse and non-human primate models [228,229], providing proof-of-principle evidence for the utility of targeting epigenetics as a possible therapy. In addition, investigating ATP-dependent chromatin remodeling complexes, such as SWItch/sucrose non-fermentable (SWI/SNF), referred to in Figure 6, may provide information on transcription and chromatin structure regulation in AMD [230,231,232,233]. This has been implied as a predictive biomarker in immune checkpoints in multiple cancers [234].

Multiple lncRNA candidates have been linked to the pathogenesis of AMD. An inhibition of lncRNA may provide protective functions, inhibiting AMD progression. For example, inhibiting LINC00167 may reduce *VEGFA* secretion and mitochondrial reactive oxygen species (ROS), thereby decreasing oxidative stress [204]. Similarly, LncRNA H-19 inhibition reduced *VEGFA* levels, macrophage markers, and neovascularization, indicating that it may act as a potential therapeutic target [235,236] (refer to Table 2). Future studies should also consider combining lncRNA inhibition with established therapeutic targets, such as anti-*VGEFA* treatments, to address multiple disease aspects, potentially resulting in more advanced control of AMD progression.

Many studies have highlighted a potential role of epigenetics in AMD and its potential use as a therapeutic target, although gaps in our understanding remain to be elucidated. As outlined in Figure 6, future investigations could strengthen knowledge on the molecular mechanism involved in AMD by developing a comprehensive genome-wide map of AMD to identify mutations and regulatory elements, such as enhancers, silencers, and super-enhancers, involved in the disease. Research into histone variants, such as H3.3, and their chaperones, may reveal novel regulatory elements involved in promoting cell survival in AMD. Epigenome editing tools, such as clustered regularly interspaced short palindromic repeats—dead Cas 9 (CRISPR-Dcas9) [237], may provide avenues for editing mutations and for investigating epigenetic modifications, such as the influence of methyltransferase and acetyltransferase on chromatin structure in the disease state [237]. Although this tool has not been applied to AMD, it could assist in validating the role of epigenetic switches in disease, supporting the development of personalized therapies.

Next-generation sequencing technology (NGS), such as ChIP-seq, ATAC-seq, and RNA-seq, can map out histone marks, provide data on the presence and location of transcription factors [155], as well as chromatin accessibility. This will assist in identifying factors involved in the early stages of disease, compared to factors involved in disease progression, potentially guiding the production of targeted therapy. Finally, a genome-wide, cell-specific epigenome map of the retina generated using spatial multi-omic approaches could link genes, environmental risk factors, and epigenetic influences on AMD pathogenic pathways [238]. This would allow for a more comprehensive understanding of the molecular mechanisms involved in the disease.

## 6. Conclusions

AMD remains a leading cause of blindness in aging populations, with limited treatment options due to its complex, multifactorial etiology. Genetic and environmental risk factors are well-established contributors to the disease. However, the underlying molecular mechanisms driving AMD pathogenesis remain unclear. Advancements in recent studies, including those that have probed DNA methylation, histone modifications, lncRNA, and chromatin remodeling, suggest that epigenetics may serve as a link between genes and environmental exposures.

This review focused on highlighting the growing evidence of epigenetic regulation across model systems and patient studies in AMD, and their potential intersection with known pathways, including inflammation, oxidative stress, mitochondrial dysregulation, and angiogenesis. While these studies tantalize the potential for biomarkers and targeted therapies with respect to epigenetic regulation in AMD, work in this area is still in its infancy. Gaps in knowledge have been addressed in this review, including the requirement of longitudinal contribution to disease onset and progression. Furthermore, advancements in single-cell epigenomics and spatial transcriptomics research may accelerate the translation of epigenetic findings into clinical interventions. Addressing these questions will be crucial to further understanding how epigenetics may contribute to AMD and how such knowledge could be harnessed for potential diagnostic or therapeutic benefit.

## Abbreviations

The following abbreviations are used in this manuscript:

5-AZA-dc	5-aza-2′-deoxycytidine
ABCA4	ATP-binding cassette subfamily A member 4
AMD	Age-related macular degeneration
ANGPTL2	Angiopoietin-like 2
ANRIL	Anti-sense noncoding RNA
APOE	Apolipoprotein E
ARMS2	Age-related maculopathy susceptibility 2
ATAC-seq	Assay for transposase accessible chromatin sequencing
BrM	Bruch’s membrane
C3	Complement 3
C5	Complement 5
C7	Complement factor 7
CD46	Cluster of differentiation 46
CETP	Cholesteryl ester transfer protein
CF1	Complement factor I
CFH	Complement factor H
ChIP-seq	Chromatin immunoprecipitation sequencing
CLU	Clusterin
CNV	Choroidal neovascularization
CRISPR-Dcas9	Clustered regularly interspaced short palindromic repeats—dead cas 9
CYTOR	Cytoskeleton regulator RNA
DNA	Deoxyribonucleic acid
DNMT1	DNA methyltransferase 1
DNMT3	DNA methyltransferase 3
EBIN	End-binding protein 3
ECM	Extracellular matrix
EMT	Epithelial to mesenchymal transition
eQTL	Expression quantitative trait locus
FRZB	Frizzled-related protein
GA	Geographic atrophy
GSTM1	Glutathione S-transferase isoform mu1
GSTM5	Glutathione S-transferase isoform mu1
GTFH4	General transcription factor IIH subunit 4.
GWAS	Genome-wide association studies
H3K27ac	Histone 3 lysine 27 acetylation
H3K27me3	Histone 3 lysine 27 trimethylation
H3K4me1	Histone 3 lysine 4 monomethylation
H3K4me3	Histone 3 lysine trimethylation
HAT	Histone acetyltransferase
HDAC	Histone Deacetylase
HDACi	Histone deacetylase inhibitors
HIF1A	Hypoxia inducible factor 1-alpha
HTRA1	High temperature requirement A serine peptidase 1
IL	Interleukin
IL17RC	Interleukin 17 receptor C
iPSC	Induced pluripotent stem cells
LIPC	hepatic lipase
lncRNA	Long noncoding RNA
LINE1	Long interspersed nuclear element-1
MALAT1	Metastasis-associated lung adenocarcinoma transcript 1
MAPK	Mitogen-activated protein kinase signaling
MEG3	Maternally expressed gene 3
miRNA	MicroRNA
MTND-2	Mitochondrially encoded NADH dehydrogenase 2
nAMD	Neovascular age-related macular degeneration
ncRNA	Noncoding RNA
NF-κB	Nuclear factor kappa B
NGFR	Nerve growth factor receptor
PRS	Polygenic risk scores
PRSS50	Protease serine 50
PTM	Post-translational modifications
PWRN2	Prader–Willi region nonprotein-coding RNA
QC	Quality control
ROS	Reactive oxygen species
RPE	Retinal pigment epithelium
scATAC-seq	Single-cell assay for transposase accessible chromatin sequencing
scRNA-seq	Single-cell RNA sequencing
SHARE-seq	Simultaneous high-throughput ATAC and RNA expression with sequencing
siRNA	Small interfering RNA
SIRT1	Silent mating type information regulation 2 homolog 1
SMAD2	Mothers against decapentaplegic homolog 2
SNARE-seq	Single-nucleus chromatin accessibility and mRNA expression sequencing
SNP	Single-nucleotide polymorphism
snRNA-seq	Single-nucleus RNA-seq
SOC3	Suppressor of cytokine signaling 3
SOD2	Superoxide dismutase 2
SW/SNF	SWItch/sucrose non-fermentable
TF	Transcription factor
TGFβ1	transforming growth factor beta-1
TLE2	Transducing-like enhancer protein 2
TNF	Tumor necrosis factor
TSA	Trichoastin A
VEGF	Vascular endothelial growth factor
VEGFA	Vascular endothelial growth factor A
XBP1	Spliced X-box binding protein 1
ZNF503-AS1	Zinc finger protein 503 antisense RNA

## References

[1] Smith W., Assink J., Klein R., Mitchell P., Klaver C.C., Klein B.E., Hofman A., Jensen S., Wang J.J., de Jong P.T.V.M. (2001). Risk factors for age-related macular degeneration: Pooled findings from three continents. Ophthalmology.

[2] Vyawahare H., Shinde P. (2022). Age-Related Macular Degeneration: Epidemiology, Pathophysiology, Diagnosis, and Treatment. Cureus.

[3] Wong W.L., Su X., Li X., Cheung C.M.G., Klein R., Cheng C.-Y., Wong T.Y. (2014). Global prevalence of age-related macular degeneration and disease burden projection for 2020 and 2040: A systematic review and meta-analysis. Lancet Glob. Health.

[4] Fleckenstein M., Keenan T.D., Guymer R.H., Chakravarthy U., Schmitz-Valckenberg S., Klaver C.C., Wong W.T., Chew E.Y. (2021). Age-related macular degeneration. Nat. Rev. Dis. Primers.

[5] Provis J.M., Penfold P.L., Cornish E.E., Sandercoe T.M., Madigan M.C. (2005). Anatomy and development of the macula: Specialisation and the vulnerability to macular degeneration. Clin. Exp. Optom..

[6] Hageman G.S., Anderson D.H., Johnson L.V., Hancox L.S., Taiber A.J., Hardisty L.I., Hageman J.L., Stockman H.A., Borchardt J.D., Gehrs K.M. (2005). A common haplotype in the complement regulatory gene factor H (HF1/CFH) predisposes individuals to age-related macular degeneration. Proc. Natl. Acad. Sci. USA.

[7] Klein R.J., Zeiss C., Chew E.Y., Tsai J.-Y., Sackler R.S., Haynes C., Henning A.K., SanGiovanni J.P., Mane S.M., Mayne S.T. (2005). Complement factor H polymorphism in age-related macular degeneration. Science.

[8] Haines J.L., Hauser M.A., Schmidt S., Scott W.K., Olson L.M., Gallins P., Spencer K.L., Kwan S.Y., Noureddine M., Gilbert J.R. (2005). Complement factor H variant increases the risk of age-related macular degeneration. Science.

[9] Lorés-Motta L., Paun C.C., Corominas J., Pauper M., Geerlings M.J., Altay L., Schick T., Daha M.R., Fauser S., Hoyng C.B. (2018). Genome-wide association study reveals variants in CFH and CFHR4 associated with systemic complement activation: Implications in age-related macular degeneration. Ophthalmology.

[10] Karimi S., Nouri H., Mahmoudinejad-Azar S., Abtahi S.-H. (2023). Smoking and environmental tobacco smoke exposure: Implications in ocular disorders. Cutan. Ocul. Toxicol..

[11] West S.K. (2021). Smoking and the risk of eye diseases. Nutritional and Environmental Influences on the Eye.

[12] Seddon J.M., Reynolds R., Shah H.R., Rosner B. (2011). Smoking, dietary betaine, methionine, and vitamin D in monozygotic twins with discordant macular degeneration: Epigenetic implications. Ophthalmology.

[13] Keeling E., Lynn S.A., Koh Y.M., Scott J.A., Kendall A., Gatherer M., Page A., Cagampang F.R., Lotery A.J., Ratnayaka J.A. (2022). A High Fat “Western-style” Diet Induces AMD-like Features in Wildtype Mice. Mol. Nutr. Food Res..

[14] Figueiredo I., Farinha C., Barreto P., Coimbra R., Pereira P., Marques J.P., Pires I., Cachulo M.L., Silva R. (2024). Nutritional Genomics: Implications for Age-Related Macular Degeneration. Nutrients.

[15] Seddon J.M., Cote J., Page W.F., Aggen S.H., Neale M.C. (2005). The US twin study of age-related macular degeneration: Relative roles of genetic and environmental influences. Arch. Ophthalmol..

[16] Millen A.E., Meyers K.J., Liu Z., Engelman C.D., Wallace R.B., LeBlanc E.S., Tinker L.F., Iyengar S.K., Robinson J.G., Sarto G.E. (2015). Association between vitamin D status and age-related macular degeneration by genetic risk. JAMA Ophthalmol..

[17] Gemenetzi M., Lotery A.J. (2014). The role of epigenetics in age-related macular degeneration. Eye.

[18] Desmettre T. (2018). Epigenetics in age-related macular degeneration (AMD). J. Fr. D’ophtalmologie.

[19] Nashine S., Nesburn A.B., Kuppermann B.D., Kenney M.C. (2019). Age-related macular degeneration (AMD) mitochondria modulate epigenetic mechanisms in retinal pigment epithelial cells. Exp. Eye Res..

[20] Devi S.M., Mahalaxmi I., Kaavya J., Chinnkulandhai V., Balachandar V. (2021). Does epigenetics have a role in age related macular degeneration and diabetic retinopathy?. Genes. Dis..

[21] Dupont C., Armant D.R., Brenner C.A. (2009). Epigenetics: Definition, mechanisms and clinical perspective. Seminars in Reproductive Medicine.

[22] Jin B., Li Y., Robertson K.D. (2011). DNA methylation: Superior or subordinate in the epigenetic hierarchy?. Genes. Cancer.

[23] Henikoff S., Smith M.M. (2015). Histone variants and epigenetics. Cold Spring Harb. Perspect. Biol..

[24] Sadakierska-Chudy A., Filip M. (2015). A comprehensive view of the epigenetic landscape. Part II: Histone post-translational modification, nucleosome level, and chromatin regulation by ncRNAs. Neurotox. Res..

[25] Wei J.-W., Huang K., Yang C., Kang C.-S. (2017). Non-coding RNAs as regulators in epigenetics. Oncol. Rep..

[26] Al Aboud N.M., Tupper C., Jialal I. (2023). Genetics, epigenetic mechanism. StatPearls.

[27] Dubey S.K., Dubey R., Prajapati S.C., Jung K., Mohan K., Liu X., Roney J., Tian W., Abney J., Giarmarco M.M. (2024). Histone deficiency and hypoacetylation in the aging retinal pigment epithelium. Aging Cell.

[28] Villota-Salazar N.A., Mendoza-Mendoza A., González-Prieto J.M. (2016). Epigenetics: From the past to the present. Front. Life Sci..

[29] Yu X., Zhao H., Wang R., Chen Y., Ouyang X., Li W., Sun Y., Peng A. (2024). Cancer epigenetics: From laboratory studies and clinical trials to precision medicine. Cell Death Discov..

[30] Ge T., Gu X., Jia R., Ge S., Chai P., Zhuang A., Fan X. (2022). Crosstalk between metabolic reprogramming and epigenetics in cancer: Updates on mechanisms and therapeutic opportunities. Cancer Commun..

[31] Park Y.J., Han S.M., Huh J.Y., Kim J.B. (2021). Emerging roles of epigenetic regulation in obesity and metabolic disease. J. Biol. Chem..

[32] Basavarajappa B.S., Subbanna S. (2021). Histone methylation regulation in neurodegenerative disorders. Int. J. Mol. Sci..

[33] Mohd Murshid N., Aminullah Lubis F., Makpol S. (2022). Epigenetic changes and its intervention in age-related neurodegenerative diseases. Cell. Mol. Neurobiol..

[34] Wang K., Liu H., Hu Q., Wang L., Liu J., Zheng Z., Zhang W., Ren J., Zhu F., Liu G.-H. (2022). Epigenetic regulation of aging: Implications for interventions of aging and diseases. Signal Transduct. Target. Ther..

[35] Saul D., Kosinsky R.L. (2021). Epigenetics of Aging and Aging-Associated Diseases. Int. J. Mol. Sci..

[36] Amini M.A., Karbasi A., Vahabirad M., Khanaghaei M., Alizamir A. (2023). Mechanistic Insight into Age-Related Macular Degeneration (AMD): Anatomy, Epidemiology, Genetics, Pathogenesis, Prevention, Implications, and Treatment Strategies to Pace AMD Management. Chonnam Med. J..

[37] Flores R., Carneiro Â., Vieira M., Tenreiro S., Seabra M.C. (2021). Age-related macular degeneration: Pathophysiology, management, and future perspectives. Ophthalmologica.

[38] Bhutto I., Lutty G. (2012). Understanding age-related macular degeneration (AMD): Relationships between the photoreceptor/retinal pigment epithelium/Bruch’s membrane/choriocapillaris complex. Mol. Asp. Med..

[39] García-Layana A., Cabrera-López F., García-Arumí J., Arias-Barquet L., Ruiz-Moreno J.M. (2017). Early and intermediate age-related macular degeneration: Update and clinical review. Clin. Interv. Aging.

[40] Khan K.N., Mahroo O.A., Khan R.S., Mohamed M.D., McKibbin M., Bird A., Michaelides M., Tufail A., Moore A.T. (2016). Differentiating drusen: Drusen and drusen-like appearances associated with ageing, age-related macular degeneration, inherited eye disease and other pathological processes. Prog. Retin. Eye Res..

[41] Schlanitz F.G., Baumann B., Kundi M., Sacu S., Baratsits M., Scheschy U., Shahlaee A., Mittermüller T.J., Montuoro A., Roberts P. (2017). Drusen volume development over time and its relevance to the course of age-related macular degeneration. Br. J. Ophthalmol..

[42] Abdelsalam A., Del Priore L., Zarbin M.A. (1999). Drusen in age-related macular degeneration: Pathogenesis, natural course, and laser photocoagulation–induced regression. Surv. Ophthalmol..

[43] Murali A., Krishnakumar S., Subramanian A., Parameswaran S. (2020). Bruch’s membrane pathology: A mechanistic perspective. Eur. J. Ophthalmol..

[44] Tisi A., Feligioni M., Passacantando M., Ciancaglini M., Maccarone R. (2021). The impact of oxidative stress on blood-retinal barrier physiology in age-related macular degeneration. Cells.

[45] Haddad S., Chen C.A., Santangelo S.L., Seddon J.M. (2006). The Genetics of Age-Related Macular Degeneration: A Review of Progress to Date. Surv. Ophthalmol..

[46] Schultz N.M., Bhardwaj S., Barclay C., Gaspar L., Schwartz J. (2021). Global Burden of Dry Age-Related Macular Degeneration: A Targeted Literature Review. Clin. Ther..

[47] Ferris F.L., Fine S.L., Hyman L. (1984). Age-related macular degeneration and blindness due to neovascular maculopathy. Arch. Ophthalmol..

[48] Ratnayaka J.A., Lotery A.J. (2020). Challenges in studying geographic atrophy (GA) age-related macular degeneration: The potential of a new mouse model with GA-like features. Neural Regen. Res..

[49] Arya M., Sabrosa A.S., Duker J.S., Waheed N.K. (2018). Choriocapillaris changes in dry age-related macular degeneration and geographic atrophy: A review. Eye Vis.

[50] Bakri S.J., Bektas M., Sharp D., Luo R., Sarda S.P., Khan S. (2023). Geographic atrophy: Mechanism of disease, pathophysiology, and role of the complement system. J. Manag. Care Spec. Pharm..

[51] Madheswaran G., Ramesh S.V., Pardhan S., Sapkota R., Raman R. (2021). Impact of living with a bilateral central vision loss due to geographic atrophy—Qualitative study. BMJ Open.

[52] Sivaprasad S., Tschosik E.A., Guymer R.H., Kapre A., Suñer I.J., Joussen A.M., Lanzetta P., Ferrara D. (2019). Living with geographic atrophy: An ethnographic study. Ophthalmol. Ther..

[53] Sacconi R., Corbelli E., Querques L., Bandello F., Querques G. (2017). A review of current and future management of geographic atrophy. Ophthalmol. Ther..

[54] Danis R.P., Lavine J.A., Domalpally A. (2015). Geographic atrophy in patients with advanced dry age-related macular degeneration: Current challenges and future prospects. Clin. Ophthalmol..

[55] Borchert G.A., Shamsnajafabadi H., Hu M.L., De Silva S.R., Downes S.M., MacLaren R.E., Xue K., Cehajic-Kapetanovic J. (2023). The Role of Inflammation in Age-Related Macular Degeneration—Therapeutic Landscapes in Geographic Atrophy. Cells.

[56] Almony A., Keyloun K.R., Shah-Manek B., Multani J.K., McGuiness C.B., Chen C.-C., Campbell J.H. (2021). Clinical and economic burden of neovascular age-related macular degeneration by disease status: A US claims-based analysis. J. Manag. Care Spec. Pharm..

[57] Schmid A., Bucher F., Liczenczias E., Maslanka Figueroa S., Müller B., Agostini H. (2022). nAMD: Optimization of patient care and patient-oriented information with the help of an internet-based survey. Graefe’s Arch. Clin. Exp. Ophthalmol..

[58] Wong T., Chakravarthy U., Klein R., Mitchell P., Zlateva G., Buggage R., Fahrbach K., Probst C., Sledge I. (2008). The natural history and prognosis of neovascular age-related macular degeneration: A systematic review of the literature and meta-analysis. Ophthalmology.

[59] Chappelow A.V., Kaiser P.K. (2008). Neovascular age-related macular degeneration: Potential therapies. Drugs.

[60] Banister K., Cook J.A., Scotland G., Azuara-Blanco A., Goulao B., Heimann H., Hernandez R., Hogg R., Kennedy C., Sivaprasad S. (2022). Non-invasive testing for early detection of neovascular macular degeneration in unaffected second eyes of older adults: EDNA diagnostic accuracy study. Health Technol. Assess..

[61] Pugazhendhi A., Hubbell M., Jairam P., Ambati B. (2021). Neovascular macular degeneration: A review of etiology, risk factors, and recent advances in research and therapy. Int. J. Mol. Sci..

[62] Ahmad A., Nawaz M.I. (2022). Molecular mechanism of VEGF and its role in pathological angiogenesis. J. Cell. Biochem..

[63] Curry B., Bylsma G., Hewitt A.W., Verma N. (2017). The VEGF treatment of AMD switch study (The vTAS Study). Asia-Pac. J. Ophthalmol..

[64] Amoaku W.M., Chakravarthy U., Gale R., Gavin M., Ghanchi F., Gibson J., Harding S., Johnston R., Kelly S., Lotery A. (2015). Defining response to anti-VEGF therapies in neovascular AMD. Eye.

[65] Boyle J., Vukicevic M., Koklanis K., Itsiopoulos C. (2015). Experiences of patients undergoing anti-VEGF treatment for neovascular age-related macular degeneration: A systematic review. Psychol. Health Med..

[66] Bressler N.M., Chang T.S., Suñer I.J., Fine J.T., Dolan C.M., Ward J., Ianchulev T. (2010). Vision-related function after ranibizumab treatment by better-or worse-seeing eye: Clinical trial results from MARINA and ANCHOR. Ophthalmology.

[67] Heier J.S., Lad E.M., Holz F.G., Rosenfeld P.J., Guymer R.H., Boyer D., Grossi F., Baumal C.R., Korobelnik J.-F., Slakter J.S. (2023). Pegcetacoplan for the treatment of geographic atrophy secondary to age-related macular degeneration (OAKS and DERBY): Two multicentre, randomised, double-masked, sham-controlled, phase 3 trials. Lancet.

[68] Wykoff C.C., Ou W.C., Brown D.M., Croft D.E., Wang R., Payne J.F., Clark W.L., Abdelfattah N.S., Sadda S.R., Group T.-A.S. (2017). Randomized trial of treat-and-extend versus monthly dosing for neovascular age-related macular degeneration: 2-year results of the TREX-AMD study. Ophthalmol. Retin..

[69] Nadeem A., Malik I.A., Shariq F., Afridi E.K., Taha M., Raufi N., Naveed A.K., Iqbal J., Habte A. (2023). Advancements in the treatment of geographic atrophy: Focus on pegcetacoplan in age-related macular degeneration. Ann. Med. Surg..

[70] Schachar I.H. (2024). Concerning Syfovre Approval for Geographic Atrophy. JAMA Ophthalmol..

[71] Danzig C.J., Khanani A.M., Loewenstein A. (2024). C5 inhibitor avacincaptad pegol treatment for geographic atrophy: A comprehensive review. Immunotherapy.

[72] Jaffe G.J., Westby K., Csaky K.G., Monés J., Pearlman J.A., Patel S.S., Joondeph B.C., Randolph J., Masonson H., Rezaei K.A. (2021). C5 inhibitor avacincaptad pegol for geographic atrophy due to age-related macular degeneration: A randomized pivotal phase 2/3 trial. Ophthalmology.

[73] Patel S.S., Lally D.R., Hsu J., Wykoff C.C., Eichenbaum D., Heier J.S., Jaffe G.J., Westby K., Desai D., Zhu L. (2023). Avacincaptad pegol for geographic atrophy secondary to age-related macular degeneration: 18-month findings from the GATHER1 trial. Eye.

[74] Mousavi M., Armstrong R.A. (2013). Genetic risk factors and age-related macular degeneration (AMD). J. Optom..

[75] Chen Y., Bedell M., Zhang K. (2010). Age-related macular degeneration: Genetic and environmental factors of disease. Mol. Interv..

[76] Deng Y., Qiao L., Du M., Qu C., Wan L., Li J., Huang L. (2022). Age-related macular degeneration: Epidemiology, genetics, pathophysiology, diagnosis, and targeted therapy. Genes. Dis..

[77] Klein M.L., Schultz D.W., Edwards A., Matise T.C., Rust K., Berselli C.B., Trzupek K., Weleber R.G., Ott J., Wirtz M.K. (1998). Age-related macular degeneration: Clinical features in a large family and linkage to chromosome 1q. Arch. Ophthalmol..

[78] Shahid H., Khan J.C., Cipriani V., Sepp T., Matharu B.K., Bunce C., Harding S.P., Clayton D.G., Moore A.T., Yates J.R. (2012). Age-related macular degeneration: The importance of family history as a risk factor. Br. J. Ophthalmol..

[79] Iyengar S.K., Song D., Klein B.E., Klein R., Schick J.H., Humphrey J., Millard C., Liptak R., Russo K., Jun G. (2004). Dissection of genomewide-scan data in extended families reveals a major locus and oligogenic susceptibility for age-related macular degeneration. Am. J. Hum. Genet..

[80] Majewski J., Schultz D.W., Weleber R.G., Schain M.B., Edwards A.O., Matise T.C., Acott T.S., Ott J., Klein M.L. (2003). Age-related macular degeneration—A genome scan in extended families. Am. J. Hum. Genet..

[81] Bhumika, Bora N.S., Bora P.S. (2024). Genetic insights into age-related macular degeneration. Biomedicines.

[82] Shughoury A., Sevgi D.D., Ciulla T.A. (2022). Molecular genetic mechanisms in age-related macular degeneration. Genes..

[83] Fritsche L.G., Igl W., Bailey J.N.C., Grassmann F., Sengupta S., Bragg-Gresham J.L., Burdon K.P., Hebbring S.J., Wen C., Gorski M. (2016). A large genome-wide association study of age-related macular degeneration highlights contributions of rare and common variants. Nat. Genet..

[84] Schick T., Lorés-Motta L., Altay L., Fritsche L.G., den Hollander A.I., Fauser S. (2020). The effect of genetic variants associated with age-related macular degeneration varies with age. Investig. Ophthalmol. Vis. Sci..

[85] Black J.R.M., Clark S.J. (2016). Age-related macular degeneration: Genome-wide association studies to translation. Genet. Med..

[86] Piri N., Kaplan H.J. (2023). Role of complement in the onset of age-related macular degeneration. Biomolecules.

[87] Nischler C., Oberkofler H., Ortner C., Paikl D., Riha W., Lang N., Patsch W., Egger S.F. (2011). Complement factor H Y402H gene polymorphism and response to intravitreal bevacizumab in exudative age-related macular degeneration. Acta Ophthalmol..

[88] Clark S.J., Higman V.A., Mulloy B., Perkins S.J., Lea S.M., Sim R.B., Day A.J. (2006). His-384 allotypic variant of factor H associated with age-related macular degeneration has different heparin binding properties from the non-disease-associated form. J. Biol. Chem..

[89] Seddon J.M., Francis P.J., George S., Schultz D.W., Rosner B., Klein M.L. (2007). Association of CFH Y402H and LOC387715 A69S with progression of age-related macular degeneration. JAMA.

[90] Laine M., Jarva H., Seitsonen S., Haapasalo K., Lehtinen M.J., Lindeman N., Anderson D.H., Johnson P.T., JärVelä I., Jokiranta T.S. (2007). Y402H polymorphism of complement factor H affects binding affinity to C-reactive protein. J. Immunol..

[91] Kubicka-Trząska A., Żuber-Łaskawiec K., Dziedzina S., Sanak M., Romanowska-Dixon B., Karska-Basta I. (2022). Genetic variants of complement factor H Y402H (rs1061170), C2 R102G (rs2230199), and C3 E318D (rs9332739) and response to intravitreal anti-VEGF treatment in patients with exudative age-related macular degeneration. Medicina.

[92] Gold B., Merriam J.E., Zernant J., Hancox L.S., Taiber A.J., Gehrs K., Cramer K., Neel J., Bergeron J., Barile G.R. (2006). Variation in factor B (BF) and complement component 2 (C2) genes is associated with age-related macular degeneration. Nat. Genet..

[93] Pilotti C., Greenwood J., Moss S.E. (2020). Functional evaluation of AMD-associated risk variants of complement factor B. Investig. Ophthalmol. Vis. Sci..

[94] Armento A., Ueffing M., Clark S.J. (2021). The complement system in age-related macular degeneration. Cell. Mol. Life Sci..

[95] Singh A., Faber C., Falk M., Nissen M.H., Hviid T.V., Sørensen T.L. (2012). Altered expression of CD46 and CD59 on leukocytes in neovascular age-related macular degeneration. Am. J. Ophthalmol..

[96] Kavanagh D., Yu Y., Schramm E.C., Triebwasser M., Wagner E.K., Raychaudhuri S., Daly M.J., Atkinson J.P., Seddon J.M. (2015). Rare genetic variants in the CFI gene are associated with advanced age-related macular degeneration and commonly result in reduced serum factor I levels. Hum. Mol. Genet..

[97] Hallam T.M., Marchbank K.J., Harris C.L., Osmond C., Shuttleworth V.G., Griffiths H., Cree A.J., Kavanagh D., Lotery A.J. (2020). Rare genetic variants in complement factor I lead to low FI plasma levels resulting in increased risk of age-related macular degeneration. Investig. Ophthalmol. Vis. Sci..

[98] Lu Z.-G., May A., Dinh B., Lin V., Su F., Tran C., Adivikolanu H., Ehlen R., Che B., Wang Z.-H. (2021). The interplay of oxidative stress and ARMS2-HTRA1 genetic risk in neovascular AMD. Vessel. Plus.

[99] Tamura H., Tsujikawa A., Yamashiro K., Akagi-Kurashige Y., Nakata I., Nakanishi H., Hayashi H., Ooto S., Otani A., Yoshimura N. (2012). Association of ARMS2 genotype with bilateral involvement of exudative age-related macular degeneration. Am. J. Ophthalmol..

[100] Yang Z., Camp N.J., Sun H., Tong Z., Gibbs D., Cameron D.J., Chen H., Zhao Y., Pearson E., Li X. (2006). A variant of the HTRA1 gene increases susceptibility to age-related macular degeneration. Science.

[101] Boulton M.E., Qi X., Kanda A., Nellissery J., Mitter S.K., Grant M.B., Swaroop A. (2012). ARMS2 association with the mitochondrial outer membrane is reduced in RPE cells exposed to oxidative stress and in AMD. Investig. Ophthalmol. Vis. Sci..

[102] Chang Y.-J., Jenny L.A., Li Y.-S., Cui X., Kong Y., Li Y., Sparrow J.R., Tsang S.H. (2023). CRISPR editing demonstrates rs10490924 raised oxidative stress in iPSC-derived retinal cells from patients with ARMS2/HTRA1-related AMD. Proc. Natl. Acad. Sci. USA.

[103] Dewan A., Liu M., Hartman S., Zhang S.S., Liu D.T., Zhao C., Tam P.O., Chan W.M., Lam D.S., Snyder M. (2006). HTRA1 promoter polymorphism in wet age-related macular degeneration. Science.

[104] Williams B.L., Seager N.A., Gardiner J.D., Pappas C.M., Cronin M.C., Amat di San Filippo C., Anstadt R.A., Liu J., Toso M.A., Nichols L. (2021). Chromosome 10q26-driven age-related macular degeneration is associated with reduced levels of HTRA1 in human retinal pigment epithelium. Proc. Natl. Acad. Sci. USA.

[105] Fan D., Kassiri Z. (2020). Biology of Tissue Inhibitor of Metalloproteinase 3 (TIMP3), and Its Therapeutic Implications in Cardiovascular Pathology. Front. Physiol..

[106] Zhang M., Zhang R., Zhao X., Ma Z., Xin J., Xu S., Guo D. (2024). The role of oxidative stress in the pathogenesis of ocular diseases: An overview. Mol. Biol. Rep..

[107] Ruan Y., Jiang S., Gericke A. (2021). Age-related macular degeneration: Role of oxidative stress and blood vessels. Int. J. Mol. Sci..

[108] Gabrielle P.-H. (2022). Lipid Metabolism and Retinal Diseases.

[109] Wang Y.-F., Han Y., Zhang R., Qin L., Wang M.-X., Ma L. (2015). CETP/LPL/LIPC gene polymorphisms and susceptibility to age-related macular degeneration. Sci. Rep..

[110] Li B., Chang F.-Y., Arunkumar R., Wan Z., Addo E.K., Bernstein P.S. (2023). Hepatic Lipase (LIPC) Knockdown Increases Macular Carotenoid Influx and Cholesterol Efflux in ARPE-19 Cells. Investig. Ophthalmol. Vis. Sci..

[111] Jacobo-Albavera L., Domínguez-Pérez M., Medina-Leyte D.J., González-Garrido A., Villarreal-Molina T. (2021). The role of the ATP-binding cassette A1 (ABCA1) in human disease. Int. J. Mol. Sci..

[112] Peters F., Ebner L.J., Atac D., Maggi J., Berger W., den Hollander A.I., Grimm C. (2022). Regulation of ABCA1 by AMD-associated genetic variants and hypoxia in iPSC-RPE. Int. J. Mol. Sci..

[113] Bao X., Zhang Z., Guo Y., Buser C., Kochounian H., Wu N., Li X., He S., Sun B., Ross-Cisneros F.N. (2021). Human RGR gene and associated features of age-related macular degeneration in models of retina-choriocapillaris atrophy. Am. J. Pathol..

[114] Ren C., Cui H., Bao X., Huang L., He S., Fong H.K., Zhao M. (2023). Proteopathy Linked to Exon-Skipping Isoform of RGR-Opsin Contributes to the Pathogenesis of Age-Related Macular Degeneration. Investig. Ophthalmol. Vis. Sci..

[115] Guo Y., Chen S., Guan W., Xu N., Zhu L., Du W., Liu Z., Fong H.K., Huang L., Zhao M. (2024). Retinal G-protein-coupled receptor deletion exacerbates AMD-like changes via the PINK1–parkin pathway under oxidative stress. FASEB J..

[116] Yu C., Robman L., He W., Woods R.L., Thao L.T.P., Wolfe R., Phung J., Makeyeva G.A., Hodgson L.A., McNeil J.J. (2024). Predictive performance of an updated polygenic risk score for age-related macular degeneration. Ophthalmology.

[117] Barnstable C.J. (2022). Epigenetics and degenerative retinal diseases: Prospects for new therapeutic approaches. Asia-Pac. J. Ophthalmol..

[118] Farkas M.H., DeAngelis M.M. (2021). Age-related macular degeneration: From epigenetics to therapeutic implications. Age-Relat. Macular Degener. Clin. Genes. Back. Patient Manag..

[119] Caputo V., Strafella C., Termine A., Fabrizio C., Ruffo P., Cusumano A., Giardina E., Ricci F., Cascella R. (2021). Epigenomic signatures in age-related macular degeneration: Focus on their role as disease modifiers and therapeutic targets. Eur. J. Ophthalmol..

[120] Liu R., Wu J., Guo H., Yao W., Li S., Lu Y., Jia Y., Liang X., Tang J., Zhang H. (2023). Post-translational modifications of histones: Mechanisms, biological functions, and therapeutic targets. MedComm.

[121] Quina A., Buschbeck M., Di Croce L. (2006). Chromatin structure and epigenetics. Biochem. Pharmacol..

[122] Jiang C., Pugh B.F. (2009). Nucleosome positioning and gene regulation: Advances through genomics. Nat. Rev. Genet..

[123] Meng H., Cao Y., Qin J., Song X., Zhang Q., Shi Y., Cao L. (2015). DNA methylation, its mediators and genome integrity. Int. J. Biol. Sci..

[124] Moore L.D., Le T., Fan G. (2013). DNA methylation and its basic function. Neuropsychopharmacology.

[125] Singal R., Ginder G.D. (1999). DNA methylation. Blood J. Am. Soc. Hematol..

[126] Rajanala K., Upadhyay A. (2024). Epigenetic switches in retinal homeostasis and target for drug development. Int. J. Mol. Sci..

[127] Cai C., Meng C., He S., Gu C., Lhamo T., Draga D., Luo D., Qiu Q. (2022). DNA methylation in diabetic retinopathy: Pathogenetic role and potential therapeutic targets. Cell Biosci..

[128] Abokyi S., To C.-H., Lam T.T., Tse D.Y. (2020). Central role of oxidative stress in age-related macular degeneration: Evidence from a review of the molecular mechanisms and animal models. Oxidative Med. Cell. Longev..

[129] Advani J., Mehta P.A., Hamel A.R., Mehrotra S., Kiel C., Strunz T., Corso-Díaz X., Kwicklis M., van Asten F., Ratnapriya R. (2024). QTL mapping of human retina DNA methylation identifies 87 gene-epigenome interactions in age-related macular degeneration. Nat. Commun..

[130] Hunter A., Spechler P.A., Cwanger A., Song Y., Zhang Z., Ying G.-s., Hunter A.K., Dezoeten E., Dunaief J.L. (2012). DNA methylation is associated with altered gene expression in AMD. Investig. Ophthalmol. Vis. Sci..

[131] Subramaniam D., Thombre R., Dhar A., Anant S. (2014). DNA methyltransferases: A novel target for prevention and therapy. Front. Oncol..

[132] Maugeri A., Barchitta M., Fallico M., Castellino N., Reibaldi M., Agodi A. (2019). Characterization of SIRT1/DNMTs functions and LINE-1 methylation in patients with age-related macular degeneration. J. Clin. Med..

[133] Wei L., Liu B., Tuo J., Shen D., Chen P., Li Z., Liu X., Ni J., Dagur P., Sen H.N. (2012). Hypomethylation of the IL17RC promoter associates with age-related macular degeneration. Cell Rep..

[134] Wang Z., Huang Y., Chu F., Liao K., Cui Z., Chen J., Tang S. (2021). Integrated Analysis of DNA methylation and transcriptome profile to identify key features of age-related macular degeneration. Bioengineered.

[135] Oliver V.F., Franchina M., Jaffe A.E., Branham K.E., Othman M., Heckenlively J.R., Swaroop A., Campochiaro B., Vote B.J., Craig J.E. (2013). Hypomethylation of the IL17RC promoter in peripheral blood leukocytes is not a hallmark of age-related macular degeneration. Cell Rep..

[136] Kenney M.C., Nashine S. (2020). Further understanding of epigenetic dysfunction of the retinal pigment epithelium in AMD. Expert. Rev. Ophthalmol..

[137] Porter L.F., Saptarshi N., Fang Y., Rathi S., Den Hollander A.I., De Jong E.K., Clark S.J., Bishop P.N., Olsen T.W., Liloglou T. (2019). Whole-genome methylation profiling of the retinal pigment epithelium of individuals with age-related macular degeneration reveals differential methylation of the SKI, GTF2H4, and TNXB genes. Clin. Epigenetics.

[138] Hirasawa M., Takubo K., Osada H., Miyake S., Toda E., Endo M., Umezawa K., Tsubota K., Oike Y., Ozawa Y. (2016). Angiopoietin-like protein 2 is a multistep regulator of inflammatory neovascularization in a murine model of age-related macular degeneration. J. Biol. Chem..

[139] Li Z., Li Y., Hou Y., Fan Y., Jiang H., Li B., Zhu H., Liu Y., Zhang L., Zhang J. (2023). Association of Plasma Vitamins and Carotenoids, DNA Methylation of LCAT, and Risk of Age-Related Macular Degeneration. Nutrients.

[140] Khan M., Shah S., Lv B., Lv Z., Ji N., Song Z., Wu P., Wang X., Mehmood A. (2023). Molecular mechanisms of Alu and LINE-1 interspersed repetitive sequences reveal diseases of visual system dysfunction. Ocul. Immunol. Inflamm..

[141] Maugeri A., Barchitta M., Mazzone M.G., Giuliano F., Basile G., Agodi A. (2018). Resveratrol Restores LINE-1 Methylation Levels by Modulating SIRT1 and DNMTs Functions in Cellular Models of Age-Related Macular Degeneration. Preprints.

[142] Merbs S.L., Khan M.A., Hackler L., Oliver V.F., Wan J., Qian J., Zack D.J. (2012). Cell-Specific DNA Methylation Patterns of Retina-Specific Genes. PLoS ONE.

[143] Karemaker I.D., Vermeulen M. (2018). Single-cell DNA methylation profiling: Technologies and biological applications. Trends Biotechnol..

[144] Camacho P., Ribeiro E., Pereira B., Varandas T., Nascimento J., Henriques J., Dutra-Medeiros M., Delgadinho M., Oliveira K., Silva C. (2023). DNA methyltransferase expression (DNMT1, DNMT3a and DNMT3b) as a potential biomarker for anti-VEGF diabetic macular edema response. Eur. J. Ophthalmol..

[145] Cui X., Zhao Q., Mahata B., Wen D., Yu-Wai-Man P., Li X. (2024). Multiomic Screening Unravels the Immunometabolic Signatures and Drug Targets of Age-Related Macular Degeneration. bioRxiv.

[146] McGinty R.K., Tan S. (2013). Histone, nucleosome, and chromatin structure. Fundamentals of Chromatin.

[147] Peterson C.L., Laniel M.-A. (2004). Histones and histone modifications. Curr. Biol..

[148] Campos E.I., Reinberg D. (2009). Histones: Annotating chromatin. Annu. Rev. Genet..

[149] Khorasanizadeh S. (2004). The nucleosome: From genomic organization to genomic regulation. Cell.

[150] Kimura H. (2013). Histone modifications for human epigenome analysis. J. Hum. Genet..

[151] Turner B.M. (1991). Histone acetylation and control of gene expression. J. Cell Sci..

[152] Struhl K. (1998). Histone acetylation and transcriptional regulatory mechanisms. Genes. Dev..

[153] Greer E.L., Shi Y. (2012). Histone methylation: A dynamic mark in health, disease and inheritance. Nat. Rev. Genet..

[154] Kouzarides T. (2002). Histone methylation in transcriptional control. Curr. Opin. Genet. Dev..

[155] Park P.J. (2009). ChIP–seq: Advantages and challenges of a maturing technology. Nat. Rev. Genet..

[156] Ruijter A.J.d., GENNIP A.H.v., Caron H.N., Kemp S., KUILENBURG A.B.v. (2003). Histone deacetylases (HDACs): Characterization of the classical HDAC family. Biochem. J..

[157] Husain S., Obert E., Singh S., Schnabolk G. (2024). Inhibition of HDAC1 and 3 in the Presence of Systemic Inflammation Reduces Retinal Degeneration in a Model of Dry Age-Related Macular Degeneration. J. Ocul. Pharmacol. Ther..

[158] Schnabolk G., Obert E., Singh S., Guzman W., Husain S. (2023). Effect of HDAC Inhibition on a Model of Dry AMD in the Presence of Systemic Inflammation. Investig. Ophthalmol. Vis. Sci..

[159] Jun J.H., Kim J.-S., Palomera L.F., Jo D.-G. (2024). Dysregulation of histone deacetylases in ocular diseases. Arch. Pharmacal Res..

[160] Wang J., Feng S., Zhang Q., Qin H., Xu C., Fu X., Yan L., Zhao Y., Yao K. (2023). Roles of histone acetyltransferases and deacetylases in the retinal development and diseases. Mol. Neurobiol..

[161] Wang J., Zibetti C., Shang P., Sripathi S.R., Zhang P., Cano M., Hoang T., Xia S., Ji H., Merbs S.L. (2018). ATAC-Seq analysis reveals a widespread decrease of chromatin accessibility in age-related macular degeneration. Nat. Commun..

[162] Luu J., Kallestad L., Hoang T., Lewandowski D., Dong Z., Blackshaw S., Palczewski K. (2020). Epigenetic hallmarks of age-related macular degeneration are recapitulated in a photosensitive mouse model. Hum. Mol. Genet..

[163] Mimura T., Kaji Y., Noma H., Funatsu H., Okamoto S. (2013). The role of SIRT1 in ocular aging. Exp. Eye Res..

[164] Zhou M., Luo J., Zhang H. (2018). Role of Sirtuin 1 in the pathogenesis of ocular disease. Int. J. Mol. Med..

[165] Karbasforooshan H., Karimi G. (2018). The role of SIRT1 in diabetic retinopathy. Biomed. Pharmacother..

[166] Zhang H., He S., Spee C., Ishikawa K., Hinton D.R. (2015). SIRT1 mediated inhibition of VEGF/VEGFR2 signaling by Resveratrol and its relevance to choroidal neovascularization. Cytokine.

[167] Jiao F., Gong Z. (2020). The beneficial roles of SIRT1 in neuroinflammation-related diseases. Oxidative Med. Cell. Longev..

[168] Yang Y., Liu Y., Wang Y., Chao Y., Zhang J., Jia Y., Tie J., Hu D. (2022). Regulation of SIRT1 and its roles in inflammation. Front. Immunol..

[169] Maugeri A., Barchitta M., Mazzone M.G., Giuliano F., Basile G., Agodi A. (2018). Resveratrol modulates SIRT1 and DNMT functions and restores LINE-1 methylation levels in ARPE-19 cells under oxidative stress and inflammation. Int. J. Mol. Sci..

[170] Sun H., Li D., Wei C., Liu L., Xin Z., Gao H., Gao R. (2024). The relationship between SIRT1 and inflammation: A systematic review and meta-analysis. Front. Immunol..

[171] Donmez G., Outeiro T.F. (2013). SIRT1 and SIRT2: Emerging targets in neurodegeneration. EMBO Mol. Med..

[172] Martin A., Tegla C.A., Cudrici C.D., Kruszewski A.M., Azimzadeh P., Boodhoo D., Mekala A.P., Rus V., Rus H. (2015). Role of SIRT1 in autoimmune demyelination and neurodegeneration. Immunol. Res..

[173] Pallàs M., Casadesús G., Smith M.A., Coto-Montes A., Pelegri C., Vilaplana J., Camins A. (2009). Resveratrol and neurodegenerative diseases: Activation of SIRT1 as the potential pathway towards neuroprotection. Curr. Neurovascular Res..

[174] Li W., Wang Y., Zhu L., Du S., Mao J., Wang Y., Wang S., Bo Q., Tu Y., Yi Q. (2021). The P300/XBP1s/Herpud1 axis promotes macrophage M2 polarization and the development of choroidal neovascularization. J. Cell. Mol. Med..

[175] Li X., He S., Zhao M. (2020). An updated review of the epigenetic mechanism underlying the pathogenesis of age-related macular degeneration. Aging Dis..

[176] Buenrostro J.D., Wu B., Chang H.Y., Greenleaf W.J. (2015). ATAC-seq: A method for assaying chromatin accessibility genome-wide. Curr. Protoc. Mol. Biol..

[177] Shang P., Daley R., Mahally E.R., Stepicheva N.A., Ghosh S., Liu H., Strizhakova A., Chowdhury O., Koontz V., Hose S.L. (2022). HDAC11 is a crucial regulator for visual cycle genes and retinal function. Investig. Ophthalmol. Vis. Sci..

[178] Hamid M.A., Moustafa M.T., Càceres-del-Carpio J., Kuppermann B.D., Kenney M.C. (2018). Effects of antiangiogenic drugs on expression patterns of epigenetic pathway genes. Ophthalmic Surg. Lasers Imaging Retin..

[179] Gemenetzi M., Lotery A. (2020). Epigenetics in age-related macular degeneration: New discoveries and future perspectives. Cell. Mol. Life Sci..

[180] Klemm S.L., Shipony Z., Greenleaf W.J. (2019). Chromatin accessibility and the regulatory epigenome. Nat. Rev. Genet..

[181] Razin S.V., Iarovaia O.V., Sjakste N., Sjakste T., Bagdoniene L., Rynditch A.V., Eivazova E.R., Lipinski M., Vassetzky Y.S. (2007). Chromatin domains and regulation of transcription. J. Mol. Biol..

[182] Grandi F.C., Modi H., Kampman L., Corces M.R. (2022). Chromatin accessibility profiling by ATAC-seq. Nat. Protoc..

[183] Smith E.N., D’Antonio-Chronowska A., Greenwald W.W., Borja V., Aguiar L.R., Pogue R., Matsui H., Benaglio P., Borooah S., D’Antonio M. (2019). Human iPSC-Derived Retinal Pigment Epithelium: A Model System for Prioritizing and Functionally Characterizing Causal Variants at AMD Risk Loci. Stem Cell Rep..

[184] Baek S., Lee I. (2020). Single-cell ATAC sequencing analysis: From data preprocessing to hypothesis generation. Comput. Struct. Biotechnol. J..

[185] Hansen K.H., Bracken A.P., Pasini D., Dietrich N., Gehani S.S., Monrad A., Rappsilber J., Lerdrup M., Helin K. (2008). A model for transmission of the H3K27me3 epigenetic mark. Nat. Cell Biol..

[186] Bae S., Lesch B.J. (2020). H3K4me1 distribution predicts transcription state and poising at promoters. Front. Cell Dev. Biol..

[187] Chen S., Lake B.B., Zhang K. (2019). High-throughput sequencing of the transcriptome and chromatin accessibility in the same cell. Nat. Biotechnol..

[188] Ma S., Zhang B., LaFave L.M., Earl A.S., Chiang Z., Hu Y., Ding J., Brack A., Kartha V.K., Tay T. (2020). Chromatin potential identified by shared single-cell profiling of RNA and chromatin. Cell.

[189] Mattick J.S., Makunin I.V. (2006). Non-coding RNA. Hum. Mol. Genet..

[190] Nemeth K., Bayraktar R., Ferracin M., Calin G.A. (2024). Non-coding RNAs in disease: From mechanisms to therapeutics. Nat. Rev. Genet..

[191] Koffler-Brill T., Noy Y., Avraham K.B. (2023). The long and short: Non-coding RNAs in the mammalian inner ear. Hear. Res..

[192] Wang K.C., Chang H.Y. (2011). Molecular mechanisms of long noncoding RNAs. Mol. Cell.

[193] Kaiser P.K., Symons R.A., Shah S.M., Quinlan E.J., Tabandeh H., Do D.V., Reisen G., Lockridge J.A., Short B., Guerciolini R. (2010). RNAi-based treatment for neovascular age-related macular degeneration by Sirna-027. Am. J. Ophthalmol..

[194] Zhang C., Owen L.A., Lillvis J.H., Zhang S.X., Kim I.K., DeAngelis M.M. (2022). AMD genomics: Non-coding RNAs as biomarkers and therapeutic targets. J. Clin. Med..

[195] Berber P., Grassmann F., Kiel C., Weber B.H. (2017). An eye on age-related macular degeneration: The role of microRNAs in disease pathology. Mol. Diagn. Ther..

[196] Wang L., Lee A.Y.W., Wigg J.P., Peshavariya H., Liu P., Zhang H. (2016). miRNA involvement in angiogenesis in age-related macular degeneration. J. Physiol. Biochem..

[197] Gemayel M.C., Bhatwadekar A.D., Ciulla T. (2021). RNA therapeutics for retinal diseases. Expert. Opin. Biol. Ther..

[198] Meola N., Pizzo M., Alfano G., Surace E.M., Banfi S. (2012). The long noncoding RNA Vax2os1 controls the cell cycle progression of photoreceptor progenitors in the mouse retina. Rna.

[199] Xu X.-D., Li K.-R., Li X.-M., Yao J., Qin J., Yan B. (2014). Long non-coding RNAs: New players in ocular neovascularization. Mol. Biol. Rep..

[200] Almalki W.H., Almujri S.S. (2024). The impact of NF-κB on inflammatory and angiogenic processes in age-related macular degeneration. Exp. Eye Res..

[201] Zhang P., Lu B., Xu F., Wang C., Zhang R., Liu Y., Wei C., Mei L. (2020). Analysis of long noncoding RNAs in choroid neovascularization. Curr. Eye Res..

[202] Zhang X., Du S., Yang D., Jin X., Zhang Y., Wang D., Wang H., Zhang Y., Zhu M. (2023). LncRNA MALAT1 knockdown inhibits the development of choroidal neovascularization. Heliyon.

[203] Zhou M., Geathers J.S., Grillo S.L., Weber S.R., Wang W., Zhao Y., Sundstrom J.M. (2020). Role of Epithelial-Mesenchymal Transition in Retinal Pigment Epithelium Dysfunction. Front. Cell Dev. Biol..

[204] Chen X., Sun R., Yang D., Jiang C., Liu Q. (2020). LINC00167 regulates RPE differentiation by targeting the miR-203a-3p/SOCS3 Axis. Mol. Ther. Nucleic Acids.

[205] Ji Y., Zuo C., Liao N., Yao L., Yang R., Chen H., Wen F. (2024). Identification of key lncRNAs in age-related macular degeneration through integrated bioinformatics and experimental validation. Aging.

[206] Yu X., Luo Y., Chen G., Liu H., Tian N., Zen X., Huang Y. (2021). Long non-coding RNA PWRN2 regulates cytotoxicity in an in vitro model of age-related macular degeneration. Biochem. Biophys. Res. Commun..

[207] Zhu W., Meng Y.-F., Xing Q., Tao J.-J., Lu J., Wu Y. (2017). Identification of lncRNAs involved in biological regulation in early age-related macular degeneration. Int. J. Nanomed..

[208] Chen X., Jiang C., Qin B., Liu G., Ji J., Sun X., Xu M., Ding S., Zhu M., Huang G. (2017). LncRNA ZNF503-AS1 promotes RPE differentiation by downregulating ZNF503 expression. Cell Death Dis..

[209] Zhu Y.-X., Yao J., Liu C., Hu H.-T., Li X.-M., Ge H.-M., Zhou Y.-F., Shan K., Jiang Q., Yan B. (2018). Long non-coding RNA MEG3 silencing protects against light-induced retinal degeneration. Biochem. Biophys. Res. Commun..

[210] Acar I.E., Galesloot T.E., Luhmann U.F.O., Fauser S., Gayán J., den Hollander A.I., Nogoceke E. (2023). Whole Genome Sequencing Identifies Novel Common and Low-Frequency Variants Associated With Age-Related Macular Degeneration. Investig. Ophthalmol. Vis. Sci..

[211] Farashi S., Abbott C.J., Ansell B.R., Wu Z., Altay L., Arnon E., Arnould L., Bagdasarova Y., Balaskas K., Chen F.K. (2024). Genetic Risk of Reticular Pseudodrusen in Age-Related Macular Degeneration: HTRA1/lncRNA BX842242.1 dominates, with no evidence for Complement Cascade involvement. medRxiv.

[212] Zhang R., Wang L., Li Y., Gui C., Pei Y., Zhou G. (2023). Roles and mechanisms of long non-coding RNAs in age-related macular degeneration. Heliyon.

[213] Sharma A., Singh N.K. (2023). Long non-coding RNAs and proliferative retinal diseases. Pharmaceutics.

[214] ElSheikh R.H., Chauhan M.Z., Sallam A.B. (2022). Current and Novel Therapeutic Approaches for Treatment of Neovascular Age-Related Macular Degeneration. Biomolecules.

[215] Lanza M., Benincasa G., Costa D., Napoli C. (2019). Clinical role of epigenetics and network analysis in eye diseases: A translational science review. J. Ophthalmol..

[216] Camacho P., Ribeiro E., Pereira B., Nascimento J., Rosa P.C., Henriques J., Barrão S., Sadio S., Quendera B., Delgadinho M. (2025). DNA Methyltransferase Expression (DNMT1, DNMT3a, and DNMT3b) as a Potential Biomarker in Age-Related Macular Degeneration. J. Clin. Med..

[217] Oliver V.F., Jaffe A.E., Song J., Wang G., Zhang P., Branham K.E., Swaroop A., Eberhart C.G., Zack D.J., Qian J. (2015). Differential DNA methylation identified in the blood and retina of AMD patients. Epigenetics.

[218] Jin Y., LI A., Zheng X., WU H., Sun G., Zhang X. (2022). Effects of 5-Aza-dC combined with chemotherapy regimens on the apoptosis of lung adenocarcinoma cells. Clin. Med. China.

[219] Samardzija M., Corna A., Gomez-Sintes R., Jarboui M.A., Armento A., Roger J.E., Petridou E., Haq W., Paquet-Durand F., Zrenner E. (2021). HDAC inhibition ameliorates cone survival in retinitis pigmentosa mice. Cell Death Differ..

[220] Trifunović D., Arango-Gonzalez B., Comitato A., Barth M., Del Amo E.M., Kulkarni M., Sahaboglu A., Hauck S.M., Urtti A., Arsenijevic Y. (2016). HDAC inhibition in the cpfl1 mouse protects degenerating cone photoreceptors in vivo. Hum. Mol. Genet..

[221] Schnichels S., Schultheiß M., Hofmann J., Szurman P., Bartz-Schmidt K.U., Spitzer M.S. (2012). Trichostatin A induces cell death at the concentration recommended to differentiate the RGC-5 cell line. Neurochem. Int..

[222] Desjardins D., Liu Y., Crosson C.E., Ablonczy Z. (2016). Histone deacetylase inhibition restores retinal pigment epithelium function in hyperglycemia. PLoS ONE.

[223] Tokarz P., Kaarniranta K., Blasiak J. (2016). Inhibition of DNA methyltransferase or histone deacetylase protects retinal pigment epithelial cells from DNA damage induced by oxidative stress by the stimulation of antioxidant enzymes. Eur. J. Pharmacol..

[224] Yang C., Ge L., Yu X., Lazarovici P., Zheng W. (2024). Artemisinin Confers Cytoprotection toward Hydrogen Peroxide-Induced Cell Apoptosis in Retinal Pigment Epithelial Cells in Correlation with the Increased Acetylation of Histone H4 at Lysine 8. Molecules.

[225] Liu S.-S., Wu F., Jin Y.-M., Chang W.-Q., Xu T.-M. (2020). HDAC11: A rising star in epigenetics. Biomed. Pharmacother..

[226] Jung K.S., Dubey R., Dubey S.K., Ashley N.F., Tian W.V., Kleinman M.E. (2023). HDAC1/2 inhibition induces cell-type dependent effects on viability and histone H3 and H4 acetylation. Investig. Ophthalmol. Vis. Sci..

[227] Kleinman M.E., Dubey R., Davila A., Dubey S.K. (2022). Acetyl-histone profiling of degenerating RPE after selective HDAC1/2 inhibition. Investig. Ophthalmol. Vis. Sci..

[228] Lee Q., Chan W.C., Qu X., Sun Y., Abdelkarim H., Le J., Saqib U., Sun M.Y., Kruse K., Banerjee A. (2023). End binding-3 inhibitor activates regenerative program in age-related macular degeneration. Cell Rep. Med..

[229] Chan M., Le J., Maienschein-Cline M., Komarova Y. (2022). EB3 inhibitor prevents AMD via widespread opening of chromatin. Investig. Ophthalmol. Vis. Sci..

[230] Sun S., Chen Y., Ouyang Y., Tang Z. (2024). Regulatory Roles of SWI/SNF Chromatin Remodeling Complexes in Immune Response and Inflammatory Diseases. Clin. Rev. Allergy Immunol..

[231] Zhang Z., Wang X., Xin J., Ding Z., Liu S., Fang Q., Yang N., Xu R.-m., Cai G. (2018). Architecture of SWI/SNF chromatin remodeling complex. Protein Cell.

[232] Chmykhalo V.K., Deev R.V., Tokarev A.T., Polunina Y.A., Xue L., Shidlovskii Y.V. (2025). SWI/SNF complex connects signaling and epigenetic state in cells of nervous system. Mol. Neurobiol..

[233] Gatchalian J., Liao J., Maxwell M.B., Hargreaves D.C. (2020). Control of stimulus-dependent responses in macrophages by SWI/SNF chromatin remodeling complexes. Trends Immunol..

[234] Wang D., Wang J., Zhou D., Wu Z., Liu W., Chen Y., Chen G., Zhang J. (2023). SWI/SNF complex genomic alterations as a predictive biomarker for response to immune checkpoint inhibitors in multiple cancers. Cancer Immunol. Res..

[235] Thomas A.A., Biswas S., Feng B., Chen S., Gonder J., Chakrabarti S. (2019). lncRNA H19 prevents endothelial–mesenchymal transition in diabetic retinopathy. Diabetologia.

[236] Sun B., Ding Y., Jin X., Xu S., Zhang H. (2019). Long non-coding RNA H19 promotes corneal neovascularization by targeting microRNA-29c. Biosci. Rep..

[237] Cai R., Lv R., Shi X.e., Yang G., Jin J. (2023). CRISPR/dCas9 tools: Epigenetic mechanism and application in gene transcriptional regulation. Int. J. Mol. Sci..

[238] Kiessling P., Kuppe C. (2024). Spatial multi-omics: Novel tools to study the complexity of cardiovascular diseases. Genome Medicine.

